# Asymptomatic Hyperuricemia Promotes Recovery from Ischemic Organ Injury by Modulating the Phenotype of Macrophages

**DOI:** 10.3390/cells11040626

**Published:** 2022-02-11

**Authors:** Viviane Gnemmi, Qiubo Li, Qiuyue Ma, Letizia De Chiara, Giulia Carangelo, Chenyu Li, Mireia Molina-Van den Bosch, Paola Romagnani, Hans-Joachim Anders, Stefanie Steiger

**Affiliations:** 1 Service d’Anatomie Pathologique, Centre de Biologie Pathologique, CHU Lille, 59037 Lille, France; viviane.gnemmi@univ-lille.fr; 2 Division of Nephrology, Department of Medicine IV, University Hospital, Ludwig-Maximilians-University Munich, 80336 Munich, Germany; qiubo.li@med.uni-muenchen.de (Q.L.); maqiuyue2014@hotmail.com (Q.M.); chenyu.li@med.uni-muenchen.de (C.L.); mireiamoli@gmail.com (M.M.-V.d.B.); hans-joachim.anders@med.uni-muenchen.de (H.-J.A.); 3 Department of Experimental and Clinical Biomedical Sciences “Mario Serio”, University of Florence, 50139 Florence, Italy; letizia.dechiara@unifi.it (L.D.C.); giulia.carangelo@unifi.it (G.C.); paola.romagnani@unifi.it (P.R.)

**Keywords:** hyperuricemia, uric acid, antioxidant, ischemia reperfusion, macrophages, tubular epithelial cells, inflammation, fatty acid oxidation, acute kidney injury

## Abstract

Acute organ injury, such as acute kidney injury (AKI) and disease (AKD), are major causes of morbidity and mortality worldwide. Hyperuricemia (HU) is common in patients with impaired kidney function but the impact of asymptomatic HU on the different phases of AKI/AKD is incompletely understood. We hypothesized that asymptomatic HU would attenuate AKD because soluble, in contrast to crystalline, uric acid (sUA) can attenuate sterile inflammation. In vitro, 10 mg/dL sUA decreased reactive oxygen species and interleukin-6 production in macrophages, while enhancing fatty acid oxidation as compared with a physiological concentration of 5 mg/dL sUA or medium. In transgenic mice, asymptomatic HU of 7–10 mg/dL did not affect post-ischemic AKI/AKD but accelerated the recovery of kidney excretory function on day 14. Improved functional outcome was associated with better tubular integrity, less peritubular inflammation, and interstitial fibrosis. Mechanistic studies suggested that HU shifted macrophage polarization towards an anti-inflammatory M2-like phenotype characterized by expression of anti-oxidative and metabolic genes as compared with post-ischemic AKI-chronic kidney disease transition in mice without HU. Our data imply that asymptomatic HU acts as anti-oxidant on macrophages and tubular epithelial cells, which endorses the recovery of kidney function and structure upon AKI.

## 1. Introduction

Acute kidney injury (AKI) and disease (AKD) are serious common public health concerns with high incidence, mortality, morbidity, and economic impact [1]. Triggers of AKI/AKD are diverse and include ischemia, sepsis, and toxic injuries [2]. AKI/AKD survivors experience irreversible losses of more or less epithelial cells and entire nephrons, which implies chronic kidney disease (CKD) with all its implications on cardiovascular health, kidney cancer, and the risk for ultimate kidney failure [2]. Independent of the primary cause, AKI/AKD is characterized by tubular epithelial cell injury and immune cell infiltration [3]. Previous reports demonstrate an essential role for macrophages on tubular epithelial cell survival and recovery in AKI/AKD, both during the injury as well as the repair and/or tissue remodeling phase [4,5]. Tissue injury and remodeling during AKI/AKD is associated with impaired metabolic functions, oxidative stress, and progressive energy waste, such as a defect in fatty acid oxidation (FAO) [6]. Notably, metabolic reprogramming via FAO and mitochondrial biogenesis can influence the secretory phenotype of macrophages [6,7,8,9]. Novel approaches to target mitochondrial biogenesis are currently under investigation, with the aim of promoting mitochondrial function or FAO to attenuate AKI–CKD transition following AKI/AKD [10].

Renal UA clearance is a marker of excretory kidney function; hence, kidney injury is usually associated with hyperuricemia (HU). Vice versa, sudden onset of severe HU may even cause AKI, e.g., in tumor lysis syndrome [11,12] and acute urate nephropathy [13], because UA forms crystals in the glomerular ultrafiltrate. However, data on a causal relationship between asymptomatic HU and AKI/AKD remain inconclusive. For example, Sanchez-Lozada et al. demonstrated that potassium oxonate-induced HU can cause AKI in rats [14], possibly by involving crystalline UA—at least this was not explicitly excluded. Further studies reported that mild HU induces apoptosis in proximal tubular epithelial cells [15] and that moderate HU induces tubular injury and macrophage infiltration in a murine model of cisplatin-induced AKI [16]. However, all in vivo studies are based on low serum UA levels of 2–5 mg/dL that are not considered as HU in humans. In vitro studies using crystalline UA (cUA) consistently report pro-inflammatory and cytotoxic effects in murine and human monocytes and macrophages [17,18,19,20,21], similar to what has been broadly reported with other crystalline particles [22,23,24]. While these effects likely attribute to UA microcrystal contaminations, recent evidence indicates that microcrystal-free soluble UA (sUA) preparations rather attenuate pro-inflammatory effects of human monocytes [25]. Whether sUA modulates resident and infiltrating tissue macrophages in AKI/AKD in the same way is unknown also because for the lack of a suitable animal model that mimics the range of human HU. To overcome this issue, we employed a novel transgenic mouse model of significant HU and induced ischemia/reperfusion injury-related AKI/AKD. We hypothesized that sUA would act as anti-oxidant by enhancing metabolic activity and driving an anti-inflammatory M2-like macrophage phenotype, as well as restoring tubular epithelial cell integrity, thus improving the recovery after acute organ injury. 

## 2. Materials and Methods

### 2.1. Animal Model of Ischemia-Reperfusion-Induced Acute Kidney Injury

All mice were housed in groups of five in filter-top cages and had access to food and water ad libitum. Cages, nest lets, food, and water were sterilized by autoclaving before use. Six-week-old Alb-creERT2;*Glut9*^lox/lox^ mice and *Glut9*^lox/lox^ control mice (kindly provided by Frederic Preitner and Bernhard Thorens, University of Lausanne, Switzerland) were injected intraperitoneally (i.p.) with tamoxifen every alternate day for one week [26,27]. Both groups of mice received a standard chow diet enriched with 25.6 g inosine per kg (Ssniff, Soest, Germany) for 3 weeks. The Alb-creERT2;*Glut9*^lox/lox^ mice developed asymptomatic HU without crystalluria and kidney injury (HU), whereas *Glut9*^lox/lox^ mice remained healthy and served as control (non-HU) [27]. Group sizes are indicated in the corresponding figure legends. 

We chose a well-established model of unilateral ischemia reperfusion injury (IRI) combined with uninephrectomy [28,29]. Briefly, Alb-creERT2;*Glut9*^lox/lox^ and *Glut9*^lox/lox^ mice were given buprenorphine (0.1 mg/kg body weight) for pain control and then anesthetized with a combination of anesthetics (fentanyl 0.05 mg/kg, midazolam 2 mg/kg, and medetomidine 0.5 mg/kg body weight) via i.p. injection and kept in a heat chamber for body temperature control. During surgical procedures, body temperature was maintained at 37 °C by placing the mice on a heating pad and monitored online using a rectal probe. One week prior to IRI, we surgically removed the right kidney (uninephrectomy). To induce ischemic injury, HU and healthy mice were subjected to unilateral renal occlusion for 20 min using nontraumatic vascular clips to clamp both the left renal artery and vein. Occlusion was verified by the color change of the left kidney from red to dark purple. After ischemia, the vascular clips were removed and reperfusion was confirmed visually by the change in kidney color to red. After wound closure, mice received antagonists (atipamezol 2.5 mg/kg and flumazenil 0.5 mg/kg body weight) and pain control with buprenorphine (0.1 mg/kg body weight). The HU and non-HU sham-operated mice underwent the same surgical procedure without unilateral kidney clamps. Regular animal welfare scoring was performed in the post-surgical period. 

Serum was collected on day 0 and before sacrifice on day 3 and 14 after IRI and stored at −20 °C until analysis. The left kidney was decapsulated and cut into three parts: one part was fixed in formalin to perform histological analysis, another fresh part was immediately used to perform flow cytometry to identify infiltrating immune cells, and the third part was stored in RNA later solution (Qiagen, Hilden, Germany) for mRNA expression analysis. 

### 2.2. Assessment of Kidney Excretory Function

Kidney function was assessed by measuring serum blood urea nitrogen (BUN) and glomerular function rate (GFR) prior to ischemia and on day 3 and 14 after reperfusion. Serum BUN was assessed enzymatically according to manufacturer’s protocols (Mouse Creatinine Assay Kit (80350, Crystal Chem, The Netherlands)). Transcutaneous GFR measurement was performed using the NIC-Kidney device as previously described [30,31]. Animals were anesthetized and GFR device attached on the back of the animals and allowed for 10 min to measure baseline fluorescent signal. After 10 min, 30 mg/mL FITC sinistrin was injected i.v. and the fluorescence signal recorded for 90 min. GFR was calculated using MPD Lab software (Medibeacon, Mannheim, Germany).

### 2.3. Assessment of Serum Uric Acid

Levels of UA in serum from mice and supernatants from in vitro cell culture experiments were assessed using the QuantiChrom^TM^ Uric Acid Assay Kit (DIUA-250) according to manufacturer’s instruction (BioAssay System) [27]. 

### 2.4. Histological Analysis

#### 2.4.1. Light Microscopy

After sacrifice, one part of the left kidney was fixed 24 h in 4% buffered formalin [32]. Formalin tissue was embedded in paraffin, and 2 μm sections were stained with Periodic acid–Schiff (PAS) to assess renal acute tubular damage and interstitial inflammation; with anti-F4/80 (dilution 1:300; Bio-Rad, rat anti-mouse, clone: CL:A3-1) to assess macrophage infiltration; with Picro sirius-red to assess interstitial fibrosis; and with CD31 (dilution 1:200; Dianova, Hamburg, Germany) to assess endothelial cell dysfunction. We scored tubular injury by assessing the percentage of tubules in the cortico-medullary junction that displayed cell necrosis, loss of the brush border, cast formation, and tubular dilation as follows: 0, none; 1, <10%; 2, 11%–25%; 3, 26%–45%; 4, 46%–75%; and 5, >76%. Loss of tubular compartment was assessed with L. tetragonolobus lectin (LT) detection [33]. To quantify inflammatory interstitial cells and interstitial fibrosis, we digitally analyzed ten cortico-medullary high-power fields (×400) using ImageJ software. 

#### 2.4.2. Electron Microscopy

Frozen kidney tissue specimens were thawed, fixed in Carson’s solution and post-fixed in 1% osmium tetroxide, dehydrated with acetone, and embedded in Epon 812. The ultrathin sections were contrasted with uranylacetate and lead citrate and were studied with a LEO EM 906 electron microscope (EM). Mitochondrial damage, including swelling of mitochondria with a loss of cristae and disruption of the mitochondrial membrane, was assessed in tubular epithelial cells and macrophages. To evaluate mitochondrial fragmentation, we determined the mitochondrial aspect ratio of 30 mitochondria per section/condition (the ratio between the major and minor axis of the ellipse equivalent to the mitochondrion) [34]. An observer blinded to the experimental condition performed all histological assessments. 

### 2.5. RNA Preparation and Real-Time Quantitative RT-qPCR

We used the Pure Link RNA Mini Kit (Thermo Fisher, Darmstadt, Germany) to extract total RNA from kidney tissue and primary macrophages cell culture stored in RNA later according to the manufacturer’s instructions. cDNA was synthesized from 1 µg of total RNA by reverse transcription polymerase chain reaction (RT-qPCR) using Superscript II reverse transcriptase (Thermo Fisher, Darmstadt, Germany) according to manufacturer’s instructions. The SYBR Green dye detection system was used for quantitative real-time qPCR on a Light Cycler 480 (Roche, Mannheim, Germany) [35]. The melting curve profiles were analyzed [27]. All gene expression values were normalized using *18s* rRNA as a housekeeping gene. All mouse primers used for amplification (300 nM) were purchased from Metabion (Martinsried, Germany) and are listed in Supplementary Table S1. 

### 2.6. Flow Cytometry Analysis

To determine the potential effects of HU and kidney injury on macrophage numbers and phenotype, kidneys were harvested on day 3 and 14 post IR injury. Single-cell suspensions were prepared using collagenase/DNase1 solution for 45 min at 37 °C. After centrifugation (1500 rpm for 5 min 4 °C, without break), cells were stained with the Live/Dead dye (Zombie NIR). Cells were resuspended in 1 mL PBS containing 0.5% BSA and 0.2 mM EDTA. Unspecific binding was blocked using FcR block (CD16/32) [36]. After washing, cells were stained with anti-mouse macrophage/myeloid cells markers CD45-PE/Cy5, F4/80-APC, CD206-FITC, CX3CR1-PE, MHCII-V450 (all from BioLegend, Fell, Germany), and anti-mouse CD11b-BV510 (BD Bioscience, Heidelberg, Germany). M2-like macrophages were identified as CD45 + CD11b + F4/80 + CX3CR1 + CD206+, whereas M1-like macrophages as CD45 + CD11b + F4/80 + CX3CR1 + CD206- [31]. Flow cytometry was performed on the BD FACSCanto II (BD, NJ, USA), and the data were analyzed using FlowJo 8.7 software (Tree Star, Ashland, OR, USA).

### 2.7. In Vitro Cell Culture Experiments

Isolation and culture of primary mouse bone marrow myeloid cells from *Glut9*^lox/lox^ mice, and their differentiation into macrophages was carried out in 10 cm dishes for 7 days with RPMI medium containing 10 ng/mL recombinant mouse macrophage colony stimulating factor (rmM-CSF) at 37 °C and 5% CO_2_. Fresh medium was added on day 2 and changed on day 5. On day 7, cells were detached using trypsin and ice-cold D-PBS. A total of 250,000 cells were seeded per well in 6-well plate or 10,000 cells were used for 96-well plates. Macrophages were primed with 50 pg/mL lipopolysaccharide (LPS) for 24 h prior to stimulation with or without 5 mg/dL or 10 mg/dL sUA (Sigma-Aldrich, Taufkirchen, Germany). Soluble UA was prepared using sodium hydroxide [25].

To mimic inflammation and ischemia/reperfusion-induced injury, cells were either stimulated with and without LPS (100 ng/mL) or cultured under hypoxic (5% O_2_) (using Galaxy O2 sensor incubator, Eppendorf™) or normoxic conditions (21% O_2_) with or without supernatants from necrotic tubular epithelial cells (necrotic soups, 20%) for 24 h then returned to reoxygenation for 24 h. Culture supernatants and RNA were collected 24 h later. For cytotoxicity and cell death, lactate dehydrogenase (LDH) release/activity was measured using the LDH assay (Roche, Mannheim, Germany) according to the manufacturer’s instructions. For enzyme-linked immunosorbent assay (ELISA), cell supernatants were collected and centrifuged to remove dead cells. The supernatants were analyzed using mouse IL-6- and IL-1β-specific ELISA kits (eBioscience, Frankfurt am Main, Germany, and Thermo Fisher, Darmstadt, Germany, respectively).

### 2.8. Oxidative Stress Analysis

To study reactive oxygen species (ROS) production, bone marrow-differentiated macrophages were primed with LPS (50 pg/mL) for 12 h and then detached by adding ice-cold D-PBS for 30 min and trypsin for 5 min at 37 °C. Cell counts were performed and 10,000 cells/well in 200 µL RPMI, 10% FCS, 1% PS, and 25 ng/mL rmM-CSF transferred into 96-well plates. The day after, medium was replaced with RPMI and cells were exposed to sUA [37] prior to hypoxia/normoxia experiments. ROS staining was achieved by incubating the cells with 100 µM of dihydrorhodamine 123 dye in the dark at 37 °C for 20 min. After washing with warm PBS and medium, fluorescence was observed under fluorescent microscope. Three fields were observed in each well, and image analysis was performed using ImageJ software. 

### 2.9. Uric Acid Uptake Assay

Mouse bone marrow-differentiated macrophages (2 × 10^6^ cells/well) were pretreated with or without 5 or 10 mg/dL sUA for 1 h in RPMI media. Cells were harvested, washed with D-PBS, and digested as previously described [25]. The concentration of sUA in cell cytosol was measured by commercial UA assay kit (BioAssay Systems, Hayward, CA, USA), and the intracellular sUA uptake rate was determined by adjusting the UA levels to cell number in each sample.

### 2.10. Analysis of Open Access Gene Expression Profiles

We analyzed expression patterns of urate reabsorption transporters *Slc2a9*, *Slc22a11*, *Slc22a12*, and *Slc22a13* and urate excretion transporters *Slc2a6*, *Slc2a7*, *Abcc4*, *Slc17a1*, and *Slc17a3* as previously described of either human blood monocyte-differentiated macrophages (*n* = 3 controls) [38] and mouse bone marrow-derived macrophages (*n* = 3 unstimulated controls) [39]. All data were downloaded as a processed expression matrix without quality control, which means that the reliability of the Affymetrix GeneChip microarray is unknown. Microarray of human macrophages (GSE162416) used the platform Illumina HumanHT-12 V4.0 expression beadchip [38] and that of mouse macrophages (GSE100059) used Affymetrix Mouse Exon 1.0 ST Array (Mm_ENTREZG_v18) [39]. Heat maps were generated using GraphPad Prism 7 software. 

### 2.11. Single Cell RNA-Sequencing

Wild-type C57Bl/6 mice underwent ischemia/reperfusion injury (IRI) at 7 weeks for 30 min as previously described [40]. Mice were sacrificed at 3 days (*n* = 3) and 7 days (*n* = 3) after IRI. Healthy mouse kidney (*n* = 1) was used as control. At the time of sacrifice, kidneys were minced into 1 mm pieces and incubated at 37 °C for 10 min in a buffer containing 250 U/mL Liberase (Roche, Mannheim, Germany) and 40 U/mL DNase I (Sigma-Aldrich, Taufkirchen, Germany). The obtained solution was then transferred to a Miltenyi C-tube, and the gentleMACS D1 program was run (Miltenyi Biotec, Bergisch Gladbach, Germany). The steps were repeated twice. The reaction was stopped by adding 10% FBS, and the dissociated cells were passed through a 40 µm cell strainer and incubated with RBC lysis buffer. Dead cells were removed employing the dead cell removal kit (Miltenyi Biotec, Bergisch Gladbach, Germany) and run on a 10x Chromium Single Cell instrument (10× Genomics). This method generated single cell suspension with greater than 95% viability.

### 2.12. Bioinformatic Analysis of Single Cell RNA-Sequencing

Raw sequencing data were processed using the 10x Genomics Cell Ranger pipeline (software version 3.0.1, Leiden, The Netherlands). First, *cellranger mkfastq* demultiplexed libraries based on sample indices converted the barcode and read data to FASTQ files. Second, *cellranger count* took FASTQ files and performed alignment to the mouse mm10 reference genome [41], respectively, and then proceeded with filtering and unique molecular identifier (UMI) counting. Next, we performed quality control on the datasets to remove poor-quality cells and badly detected genes. Specifically, we filtered out cells with a mitochondrial read >40% and <200 genes, respectively. Cell-specific biases were normalized by dividing the measured counts by the size factor obtained through the scran *computeSumFactors* method [42]. Finally, all counts were log-transformed after addition of a pseudocount of 1. After quality control and filtering, we obtained 3396 cells from healthy mouse kidney and 2751 cells and 1845 cells from IRI at day 3 and 7, respectively.

Next, we mitigated the batch effect through the *pp.mnn_correct* function [43] to later proceed with feature selection to keep “informative” genes only used for dimensional reduction through principal component analysis (PCA). The first 50 principal components (PCs) were used to construct a neighborhood graph of observations (Leland McInnes, J.H., James Melville. UMAP: Uniform Manifold Approximation and Projection for Dimension Reduction. arXiv.org (2018)) through the *pp.neighbors* function, which relies on the Uniform Manifold Approximation and Projection (UMAP) algorithm to estimate the connectivity of data points. The data were clustered at resolutions (0.5, 1, 2) by the *tl.louvain* function.

### 2.13. Statistical Analysis

Statistical analysis was performed using GraphPad Prism 7 software (La Jolla, CA, USA). Data were checked for normal distribution by Kolmogorov–Smirnov test and compared either by Student’s *t*-test to calculate significance between two groups or between three or more groups using one-way ANOVA with Tukey’s post hoc test. For two parameters with multiple groups, we used two-way ANOVA with Bonferroni’s comparison post hoc test. Data are presented as mean ± SEM. Differences were considered significant if *p* < 0.05; ns indicates not significant. Sample/group sizes are indicated in each corresponding figure legend.

## 3. Results

### 3.1. Soluble Uric Acid Inhibits the Pro-Inflammatory Function of Activated Macrophages and Increases Fatty Acid Oxidation 

To investigate whether sUA can exert anti-inflammatory and anti-oxidative effects in macrophages, we first carried out in vitro experiments with bone marrow-differentiated macrophages in the absence or presence of sUA (5 mg/dL = normouricemia; 10 mg/dL = HU) prior to stimulation with LPS (Figure S1A). The UA concentrations in the supernatants after stimulation correlated with the sUA concentrations of 5 and 10 mg/dL added to the macrophage culture (Figure S1B). Stimulating macrophages with LPS induced an increase in the production of ROS, as indicated by the fluorescence signal of dihydrorhodamine 123 as compared with medium, an effect that was reduced in the presence of 10 mg/dL sUA (Figure S1C,D). Similar results were observed with the production of IL-6 and IL-1β in activated macrophages in the presence of sUA (Figure S1E,F), suggesting an anti-inflammatory effect of sUA. 

**Figure 1:**
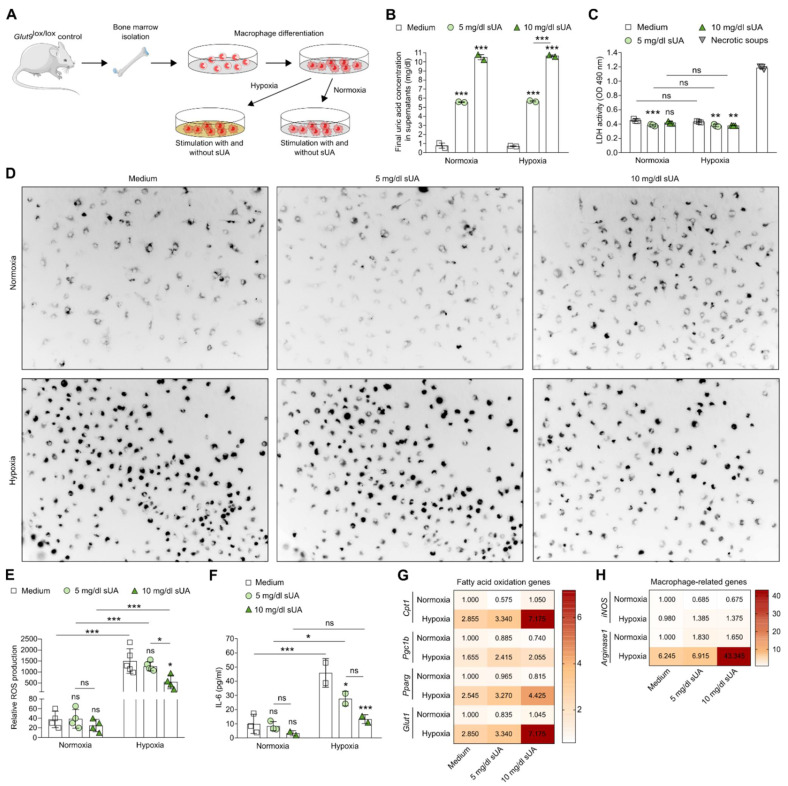
Soluble uric acid inhibits pro-inflammatory functions and enhances the metabolic activity of macrophages under hypoxic conditions. (**A**) Schematic of experimental setup. Bone marrow-derived macrophages from *Glut9*^lox/lox^ mice were primed with LPS for 24 h prior to sUA stimulation (medium; 5 mg/dL sUA; 10 mg/dL sUA). To mimic ischemic/reperfusion, cells were cultured under hypoxic (5% O_2_) and/or normoxic (21% O_2_) conditions for 24 h. (**B**) Final UA concentrations after adding 5 and 10 mg/dL sUA into the macrophage culture were measured by colorimetric assay. (**C**) Cytotoxicity (LDH activity) of macrophages was assessed by LDH assay (absorbance, OD 490 nm) (*n* = 3). DAMP-containing supernatants from necrotic tubular epithelial cells were used as positive control (necrotic soups). (**D**,**E**) Representative images (**D**) and quantification of ROS production (**E**) was detected with dihydrorhodamine 123 dye using a fluorescent microscope (*n* = 4–5). (**F**) Concentrations of interleukin (IL)-6 in culture supernatants were determined by ELISA (*n* = 2–3). (**G**) mRNA expression of genes associated with fatty acid oxidation *Cpt1*, *Pgc1b*, *Pparg*, and *Glut1* was determined by RT-qPCR (*n* = 4–5). (**H**) mRNA expression of the M1-like pro-inflammatory gene *iNos* and the M2-like anti-inflammatory gene *Arg-1* was determined by RT-qPCR (*n* = 4–5). Data are expressed as mean ± SEM. * *p* < 0.05; ** *p* < 0.01; *** *p* < 0.001; ns, not significant; two-way ANOVA with Tukey post hoc test.

Much research has focused on the impact of hypoxia on macrophage polarization and activation in tumor biology [44,45]. Hypoxia also modulates inflammation in cardiovascular diseases, rheumatoid arthritis, and ischemic injury, e.g., in AKI/AKD, by driving inflammation, oxidative stress, metabolic shift to glycolysis, and pro-inflammatory macrophage polarization [46]. However, whether sUA can influence macrophage functions under hypoxic conditions is currently unclear. Hence, we cultured bone marrow-differentiated macrophages in the absence or presence of sUA (5 mg/dL = normouricemia; 10 mg/dL = HU) under hypoxia to mimic ischemia more closely or normoxic conditions (Figure 1A). Hypoxia did not alter UA concentrations in the supernatants as compared with the sUA concentrations of 5 and 10 mg/dL added to the macrophage culture (Figure 1B). In addition, sUA was not cytotoxic to macrophages and rather decreased the LDH activity compared with medium under normoxic and hypoxic conditions (Figure 1C). While hypoxia induced an increase in the production of ROS in untreated macrophages (medium) as compared with normoxia, sUA (10 mg/mL) inhibited this hypoxia-related release of ROS (Figure 1D,E). Similar results were observed with IL-6 production in sUA-treated macrophages following hypoxia (Figure 1F). Hypoxia induced an increase in mRNA expression levels of FAO genes, including carnitine palmitoyl-transferase 1 (*Cpt1*), peroxisome proliferator-activated receptors gamma (*Pparg*), and Pparg coactivator-1b (*Pgc1b*), as well as glycolysis and glucose transporter 1 (*Glut1*) in untreated macrophages (medium) (Figure 1G), while sUA further enhanced mRNA expression levels of these FAO-related genes in macrophages. This was also associated with a significant increase in the M2-like macrophage marker *Arginase1* (*Arg1*) in the presence of 10 mg/dL sUA but not that of the pro-inflammatory M1-like macrophage marker *iNos* (Figure 1H). 

**Figure 2:**
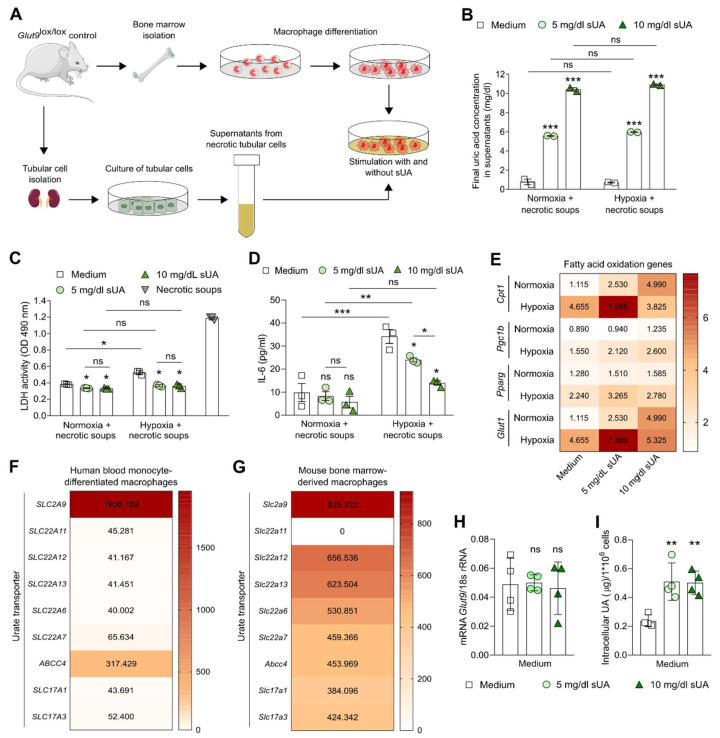
Soluble uric acid inhibits the pro-inflammatory function but enhances the metabolic activity of macrophages under hypoxic and DAMP-related tubular injury condition. (**A**) Schematic of experimental setup. Bone marrow-derived macrophages from *Glut9*^lox/lox^ mice were primed with LPS for 24 h prior to sUA stimulation (medium; 5 mg/dL sUA; 10 mg/dL sUA). To mimic ischemic/reperfusion injury in the kidney, cells were cultured under hypoxic (5% O_2_) and/or normoxic (21% O_2_) conditions with and without damage-associated molecular pattern (DAMP)-containing supernatants from necrotic tubular epithelial cells for 24 h. (**B**) Final UA concentrations after adding 5 and 10 mg/dL sUA into the macrophage culture were measured by colorimetric assay. (**C**) Cytotoxicity (LDH activity) of macrophages was assessed by LDH assay (absorbance, OD 490 nm) (*n* = 3). DAMP-containing supernatants from necrotic tubular epithelial cells were used as positive control (necrotic soups). (**D**) Concentrations of interleukin (IL)-6 in culture supernatants were determined by ELISA (*n* = 2–3). (**E**) mRNA expression levels of genes associated with fatty acid oxidation *Cpt1*, *Pgc1b*, *Pparg,* and *Glut1* were determined by RT-qPCR (*n* = 4–5). Data are expressed as mean ± SEM. (**F**,**G**) Online available microarray data of urate transporters expressed by human blood monocyte-differentiated macrophages (**F**, *n* = 3) and mouse bone marrow-derived macrophages (**G**, *n* = 3) [38,39]. (**H**) mRNA expression of the UA transporter *Slc2a9/Glut9* in mouse bone marrow-derived macrophages in the presence or absence of sUA determined by RT-PCR (*n* = 3). (**I**) Mouse bone marrow-derived macrophages with or without 5 or 10 mg/dL sUA were digested and the intracellular sUA concentration was measured (*n* = 4). * *p* < 0.05; ** *p* < 0.01; *** *p* < 0.001; ns, not significant; one- or two-way ANOVA with Tukey post hoc test.

Next, to better understand the effects of sUA on macrophage functions during ischemia/reperfusion injury, we expanded our in vitro experiments of normoxia and hypoxia by stimulating sUA-treated or untreated bone marrow-differentiated macrophages in the presence of damage-associated molecular pattern (DAMP)-containing supernatants from necrotic tubular epithelial cells (necrotic soups) to better mimic the microenvironment during AKI/AKD (Figure 2A). The UA concentrations in the supernatants did not change between the groups independent of the presence of sUA and necrotic soups in the macrophage culture under normoxic and hypoxic conditions (Figure 2B). In addition, sUA did not induce cell death in macrophages stimulated with necrotic soups but rather decreased the LDH activity compared with medium under normoxic and hypoxic conditions (Figure 2C). Hypoxia induced an increase in the production of IL-6 in necrotic soup-activated macrophages (medium) as compared with normoxia (Figure 2D). Interestingly, sUA (10 mg/mL) inhibited this hypoxia-related release of IL-6 in necrotic soup-activated macrophages (Figure 1D). Under normoxic conditions, sUA induced an increase in mRNA expression levels of the FAO genes *Cpt1*, *Pparg*, *Pgc1b,* and *Glut1* as compared with untreated macrophages (medium) in the presence of necrotic soups (Figure 2E). The mRNA expression of these FAO-related genes further increased in sUA-treated activated macrophages under hypoxia (Figure 2E), suggesting that sUA inhibits the pro-inflammatory functions of DAMP-activated macrophages, while promoting FAO in hypoxia.

We then asked the question: How could sUA inhibit the inflammatory function of macrophages? Recent evidence suggests that monocytes express urate transporters for the intracellular uptake of sUA [25]. To investigate whether this is also the case in macrophages, we searched online databases for urate transporters expressed by macrophages. Microarray data of human blood monocyte-differentiated macrophages [38] (Figure 2F) and mouse bone marrow-derived macrophages [39] (Figure 2G) from the public domain suggested that these cells highly expressed the urate transporter solute carrier family 2 member (*SLC2A9/Glut9*). RT-qPCR confirmed the expression of *SLC2A9/Glut9* in mouse bone marrow-derived macrophages (Figure 2H). Exposing macrophages to 5 or 10 mg/dL sUA for 1 h resulted in an increase in intracellular sUA concentration (Figure 2I), suggesting that *SLC2A9/Glut9*-mediated intracellular uptake of sUA might be an essential step in UA-related inhibition of activated macrophages.

### 3.2. Asymptomatic Hyperuricemia Improves Kidney Function upon Acute Organ Injury

**Figure 3:**
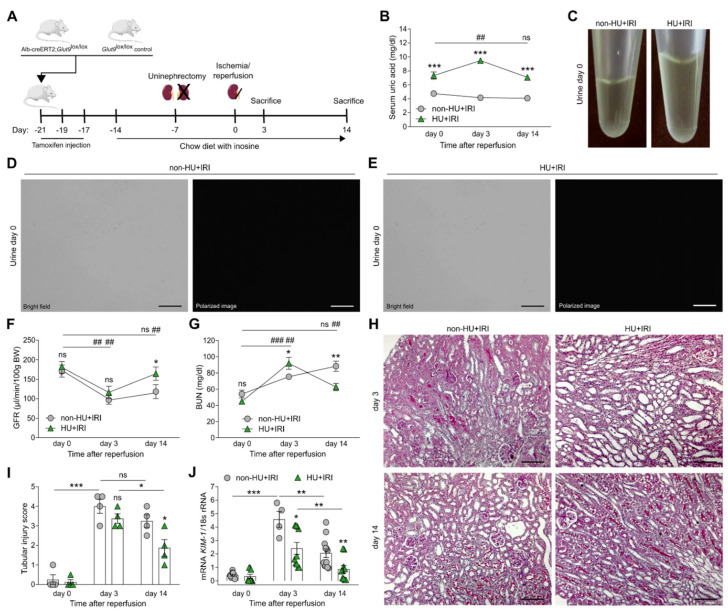
Asymptomatic hyperuricemia improves kidney function in acute organ injury. (**A**) Schematic of experimental setup. Hyperuricemic Alb-creERT2;*Glut9*^lox/lox^ (HU) and *Glut9*^lox/lox^ control (non-HU) mice were injected with tamoxifen and placed on a chow diet enriched with inosine to induce hyperuricemia (HU). Nephrectomy was performed (day 7) prior to clamping of the left renal pedicle for 15 min to induce ischemia/reperfusion injury (IRI). Mice were sacrificed on day 3 or 14. (**B**) Serum uric acid levels were measured by colorimetric assay. (**C**) Images of urine from mice before IRI showing no UA crystal deposits. (**D**,**E**) Representative bright field and polarized images of urine from non-HU + IRI and HU + IRI mice showing no UA crystals. Magnification ×400. (**F**,**G**) Kidney function was evaluated by glomerular filtration rate (GFR) (**F**) and blood urea nitrogen (BUN) levels (**G**) over time. (**H**,**I**) Representative images of PAS-stained kidney sections (**H**) and histological tubular injury scores (**I**). Magnification, ×100. (**J**) Intrarenal mRNA expression levels of the kidney injury marker KIM-1 determined by RT-qPCR. Data are mean ± SEM from 3 to 7 mice in each group. * *p* < 0.05; ** *p* < 0.01; *** *p* < 0.001; ns, not significant; ## *p* < 0.01; ### *p* < 0.001; two-way ANOVA with Tukey’s post hoc test.

To investigate a potential anti-oxidant effect of asymptomatic HU in vivo, we induced postischemic AKI/AKD in hyperuricemic Alb-creERT2;*Glut9*^lox/lox^ mice and normouricemic *Glut9*^lox/lox^ control mice and evaluated kidney function, tubular injury, inflammation, and interstitial fibrosis on day 3 and 14 (Figure 3A). AKI increased serum UA levels from 7 to 10 mg/dL in hyperuricemic Alb-creERT2;*Glut9*^lox/lox^ mice on day 3, which returned to 7 mg/dL after 14 days, while *Glut9*^lox/lox^ mice had normal serum UA levels at all time points (Figure 3B). Alb-creERT2;*Glut9*^lox/lox^ and *Glut9*^lox/lox^ mice on inosine-enriched diet did not show urinary UA crystals (Figure 3C), as illustrated by light and polarized microscopy in non-hyperuricemic (non-HU + IRI) and hyperuricemic (HU + IRI) mice (Figure 3D,E), unlike mice on an inosine-enriched acidogenic diet [27]. IRI induced a significant drop in GFR (Figure 3F) as well as an increase in serum BUN levels (Figure 3G) in both groups of mice on day 3. However, kidney function fully recovered in hyperuricemic mice (HU + IRI), as documented by the reconstitution of baseline GFR and decline in serum BUN levels on day 14, while these parameters remained abnormal in non-HU + IRI controls (Figure 3F,G). Histological analysis of PAS-stained kidney sections revealed diffuse tubular injury, outer medulla necrosis, intraluminal casts, and tubular dilation after IRI in both groups of mice on day 3, while HU mice displayed significantly less tubular injury on day 14 (Figure 3H,I) without affecting glomerular and peritubular endothelial cell integrity (Figure S2A,B). This was in line with mRNA expression levels of the kidney injury marker *KIM-1* showing significantly lower *KIM-1* mRNA expression levels in HU mice as compared with non-HU mice after IRI on days 3 and 14 (Figure 3J). 

### 3.3. Asymptomatic Hyperuricemia Restores Tubular Integrity and Enhances Fatty Acid Oxidation and Mitochondria Dynamics after Acute Organ Injury 

**Figure 4:**
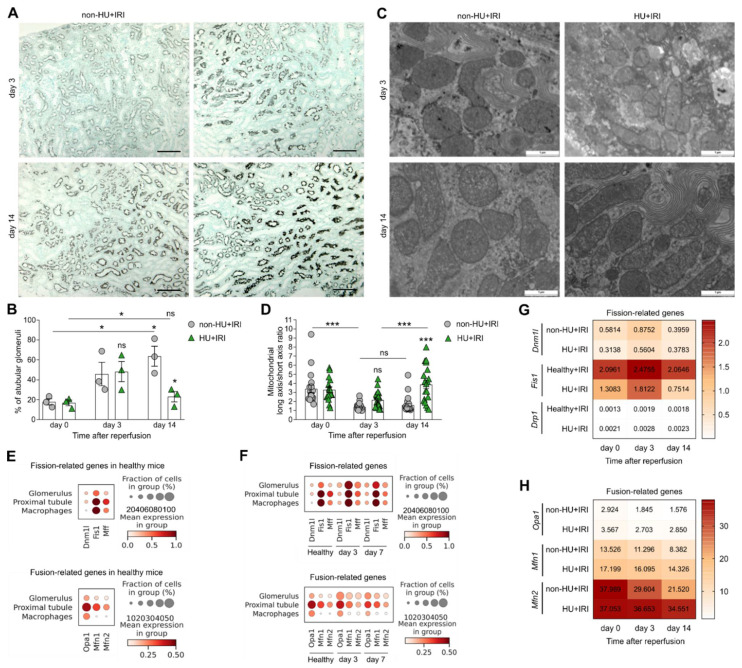
Asymptomatic hyperuricemia restores tubular integrity in acute organ injury. Hyperuricemic Alb-creERT2;*Glut9*^lox/lox^ (HU) and *Glut9*^lox/lox^ control (non-HU) mice were injected with tamoxifen and placed on a chow diet enriched with inosine to induce hyperuricemia (HU). Nephrectomy was performed (day 7) prior to clamping of the left renal pedicle for 15 min to induce ischemia/reperfusion injury (IRI). Mice were sacrificed on day 3 or 14. (**A**) Representative images of *Lotus tetragonolobus* (LT) lectin staining of proximal tubule epithelial cells of kidney sections in non-HU + IRI and HU + IRI mice on day 3 and 14. Magnification, ×100. (**B**) Percentage (%) of atubular glomeruli quantified on PAS-stained kidney sections. (**C**,**D**) Electron microscopy of mitochondria morphology in tubular epithelial cells (**C**) and quantification of the mitochondrial long axis/short axis ratio (*n* = 15–25 mitochondria, **D**). (**E**,**F**) Single cell RNA-sequencing identified mitochondria fission-related genes (*Dnm1l*, *Fis1*, *Mff*) and fusion related genes (*Opa1*, *Mfn1*, *Mfn2*) to be expressed in macrophages, proximal tubular epithelial, and glomerular cells in kidney samples from healthy mice (**E**, *n* = 1) and mice at 3 and 7 days after IRI (**F**, *n* = 3 per group). (**G**,**H**) Intrarenal mRNA expression levels of the fission-related genes *Dnm1*, *Fis1,* and GTPase dynamin-related protein 1 (*Drp1*) (**G**) and the fusion-related genes *Opa1*, *Mfn1*, and *Mfn2* from non-HU + IRI and HU + IRI mice (*n* = 3–6) determined by RT-qPCR. Data are mean ± SEM from 2–5 mice per group. * *p* < 0.05; *** *p* < 0.001; ns, not significant; two-way ANOVA with Tukey’s post hoc test.

To investigate the effects of asymptomatic HU on tubular epithelial cell function in more detail, we performed immunohistochemistry and electron microscopy of kidney sections and found that the area of recovered LT+ proximal tubular cells was higher in HU + IRI mice as compared with non-HU + IRI mice on day 3 and 14 (Figure 4A). Consistently, the percentage of atubular glomeruli was significantly lower in HU + IRI mice, as compared with non-HU + IRI mice on day 14 (Figure 4B), indicating less proximal tubular atrophy between days 3 and 14 in HU mice. Thus, asymptomatic HU protects injured tubules from atrophy and retains a higher number of functional nephrons, which explains better the kidney functional parameters in these mice compared with controls (Figure 3F,G). 

Previous studies reported that tubular mitochondrial dysfunction contributes to oxidative stress, persistent energy depletion, and cell death in AKI [47,48,49]. Restoring mitochondrial dynamics can ameliorate AKI/AKD [50,51]. To explore whether HU also restores tubular mitochondria dynamics, we performed electron microscopy of tubular mitochondria. The data revealed that upon kidney injury, the mitochondrial long axis/short axis ratio decreased in both groups of mice on day 3 as compared with day 0 (Figure 4C,D). While the morphology of tubular mitochondria remained unaffected in non-HU + IRI mice on day 3 and 14, we observed a significant increase in elongated tubular mitochondria in HU + IRI mice on day 14 as compared with non-HU + IRI mice (Figure 4C,D). In addition, we performed single cell RNA-sequencing on kidney samples obtained from mice at 3 and 7 days after IRI-related AKI and looked at cluster distribution and gene expression of mitochondria fission- and fusion-related genes (Figure S3A–D). We found that macrophages and proximal tubular epithelial and glomerular cells from healthy mice expressed the mitochondria fission-related genes dynamin 1-like (*Dnm1l*), mitochondrial fission protein 1 (*Fis1*), and mitochondrial fission factor (*Mff*), as well as the fusion-related genes optic atrophy factor 1 (*Opa1*), mitofusion 1 (*Mfn1*), and mitofusion 2 (*Mfn2*) (Figure 4E). Upon IRI, the expression pattern of fission-related genes increased, while that of fusion-related genes decreased (Figure 4F). A similar trend in the fission- and fusion-related gene expression profiles was observed in whole kidney samples from non-HU + IRI mice, as quantified by RT-qPCR (Figure 4G,H). Interestingly, mice with asymptomatic HU had reduced expression levels of fission-related genes (Figure 4G) but increased fusion-related genes (Figure 4H) as compared with non-HU + IRI mice after IRI. Taken together, asymptomatic HU can restore tubular integrity by enhancing tubular mitochondrial dynamics, in particular mitochondria fusion, that contributes to the recovery of AKI/AKD. 

**Figure 5:**
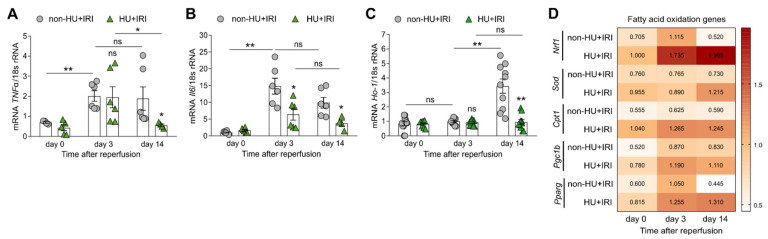
Asymptomatic hyperuricemia decreases inflammation but increases fatty acid oxidation in acute organ injury. Hyperuricemic Alb-creERT2;*Glut9*^lox/lox^ (HU) and control *Glut9*^lox/lox^ (non-HU) mice were injected with tamoxifen and placed on a chow diet enriched with inosine to induce hyperuricemia (HU). Nephrectomy was performed (day 7) prior to clamping of the left renal pedicle for 15 min to induce ischemia/reperfusion injury (IRI). Mice were sacrificed on day 3 or 14. (**A**,**B**) Intrarenal mRNA expression levels of the inflammation marker tumor necrosis factor (Tnf)-α (**A**) and interleukin (Il)-6 (**B**) in both groups of mice determined by RT-qPCR. (**C**) Intrarenal mRNA expression levels of the pro-oxidant marker heme oxygenase (Ho)-1 in both groups of mice determined by RT-qPCR. (**D**) Intrarenal mRNA expression levels of fatty acid oxidation-related genes Nrf1, Sod, Cpt1, Pparg, and Pgc1b determined by RT-qPCR. Data are mean ± SEM from 3 to 10 mice per group. * *p* < 0.05; ** *p* < 0.01; ns, not significant; two-way ANOVA with Tukey’s post hoc test.

Tubular injury in AKI/AKD is associated with inflammation [29,52]. Looking at intrarenal mRNA expression levels of inflammatory markers, we confirmed that IRI induced an increase in tumor necrosis factor (*Tnf*)-*α* and interleukin (*Il*)-*6* gene expression in non-HU mice after IRI (Figure 5A,B), while HU reduced the expression levels of both markers (Figure 5A,B), suggesting that asymptomatic HU inhibits the inflammatory response after IRI.

Soluble UA is known for its anti-oxidative activities [53]. To look for a potential anti-oxidative role of asymptomatic HU in AKI/AKD, we performed RT-qPCR for known pro-oxidant and anti-oxidant markers and found that the intrarenal mRNA expression level of the pro-oxidant heme oxygenase (*Ho*)-*1* increased on day 14 as compared with day 0 and 3 in non-HU mice after IRI (Figure 5C). However, mice with asymptomatic HU had significantly reduced mRNA expression levels of *Ho-1* after IRI on day 14 (Figure 5C). As shown in Figure 5D, asymptomatic HU enhanced the anti-oxidative response in mice after IRI (HU + IRI), as indicated by an increase in mRNA expression levels of the FAO-related genes *Cpt1*, *Pparg*, *Pgc1b*, nuclear respiratory factor 1 (*Nrf1*), and superoxide dismutase (*Sod*) compared with non-HU + IRI mice. The data suggested that asymptomatic HU acts as anti-oxidant by inducing a metabolic shift, which promotes the resolution of inflammation and tissue repair after AKI/AKD. 

### 3.4. Asymptomatic Hyperuricemia Drives an Anti-Inflammatory M2-like Macrophage Phenotype in Acute Organ Injury

**Figure 6:**
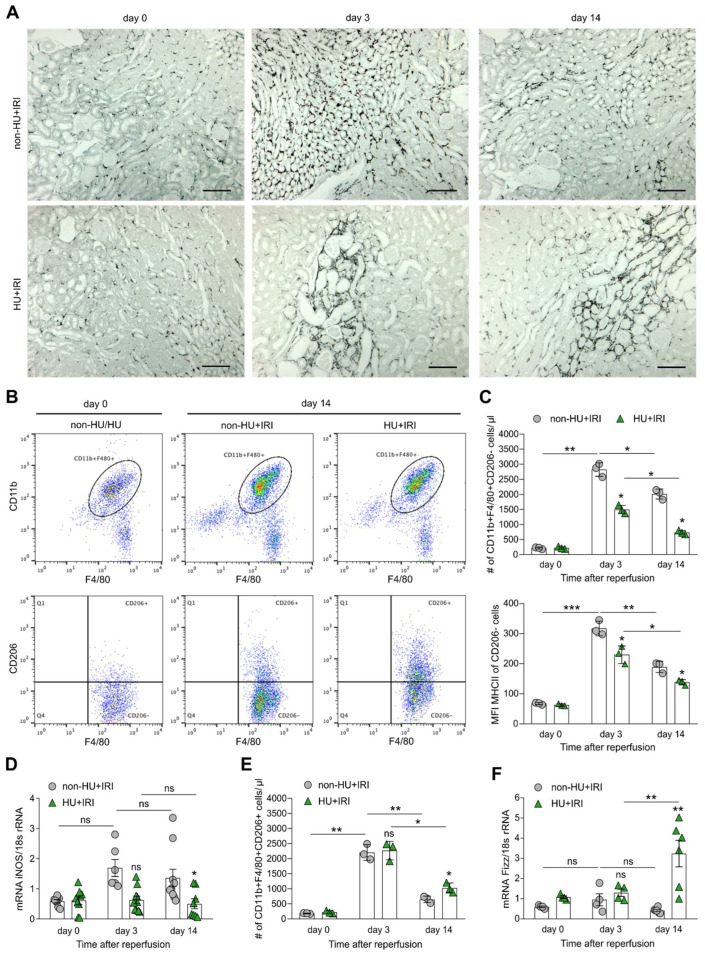
Asymptomatic hyperuricemia drives an anti-inflammatory macrophage phenotype in acute organ injury. Hyperuricemic Alb-creERT2;*Glut9*^lox/lox^ (HU) and *Glut9*^lox/lox^ control (non-HU) mice were injected with tamoxifen and placed on a chow diet enriched with inosine to induce hyperuricemia (HU). Nephrectomy was performed (day 7) prior to clamping of the left renal pedicle for 15 min to induce ischemia/reperfusion injury (IRI). Mice were sacrificed on day 3 or 14. (**A**) Immunohistochemistry of F4/80+ macrophages in kidney sections in both groups of mice at different time points. Magnification ×100. (**B**) Flow cytometry analysis with gating strategy identified macrophages as CD45 + CD11b + F4/80 + Cx3CR1+ and pro-inflammatory M1-like macrophages as CD45 + CD11b + F4/80 + Cx3CR1 + CD206- but anti-inflammatory M2-like macrophages as CD45 + CD11b + F4/80 + Cx3CR1 + CD206+ in both groups of mice. (**C**) Number of pro-inflammatory M1-like macrophages and the expression level (mean fluorescence intensity, MFI) of MHCII. (**D**) Intrarenal mRNA expression of the M1-like marker *iNos* determined by RT-qPCR. (**E**) Number of anti-inflammatory M2-like macrophages determined by flow cytometry. (**F**) Intrarenal mRNA expression of the M2-like marker *Fizz* determined by RT-qPCR. Data represent mean ± SEM from *n* = 3–8 mice in each group. * *p* < 0.05; ** *p* < 0.01; *** *p* < 0.001; ns, not significant; two-way ANOVA with Tukey’s post hoc test.

One feature of AKI/AKD-induced inflammation is the infiltration of immune cells including monocytes and macrophages, wherein the polarization of different macrophage phenotypes strongly depends on the kidney microenvironment [54] and their metabolic activity. In AKI to CKD transition, persistent macrophage infiltrates contribute in various ways to healing process and tissue remodeling. To investigate whether asymptomatic HU may influence macrophage polarization, we performed immunohistochemistry, electron microscopy, and extensive flow cytometry analysis. Immunohistochemistry of kidney sections revealed persistent tubulointerstitial infiltrates of F4/80+ cells in non-HU mice after IRI, while less macrophage infiltration was observed in HU mice on day 3 (Figure 6A). However, the number of F4/80+ macrophages decreased in both groups of mice on day 14 (Figure 6A). Further analysis using flow cytometry showed more infiltrating macrophages (CD45 + CD11b + F4/80+) in both groups of mice after IRI on day 3 and 14 as compared with non-HU mice on day 0 (Figure 6B), of which pro-inflammatory M1-like macrophages (CD45 + CD11b + F4/80 + Cx3CR1 + CD206-) and anti-inflammatory M2-like macrophages (CD45 + CD11b + F4/80 + Cx3CR1 + CD206+) were identified (Figure 6B). We found that the number of M1-like macrophages was significantly lower in HU mice (HU + IRI) as compared with non-HU mice after IRI on day 3 and 14, which was associated with a decrease in MHCII expression, as indicated by a lower mean fluorescence intensity (MFI) (Figure 6C) as well as mRNA expression levels of the pro-inflammatory macrophage marker *iNos* (Figure 6D). In contrast, we observed an increase in the number of M2-like macrophages in HU mice after IRI on day 14 as compared with non-HU mice but not on day 3 (Figure 6E). This was in line with an increase in the mRNA expression levels of the anti-inflammatory macrophage marker *Fizz* in HU mice after IRI on day 14 (Figure 6F).

Mitochondria are also key for energy metabolism in macrophages and the regulation of macrophage biology [55,56]. For instance, M1-like macrophages are characterized by increased glycolytic metabolism, production of pro-inflammatory mediators (e.g., ROS, IL-6), and mitochondria fragmentation (fission), while M2-like macrophages display increased FAO and release of anti-inflammatory cytokines, as well as mitochondria elongation/fusion [57]. To investigate whether asymptomatic HU can cause structural changes in mitochondria in macrophages, we carried out electron microscopy of macrophage mitochondria of kidney sections from non-HU and HU mice after IRI and found that HU changed the morphological features of mitochondria in macrophages, as indicated by an increase in the macrophage mitochondria long/short axis ratio (more elongated mitochondria, less fragmented) as compared with macrophages from non-HU + IRI mice (Figure S4). Together, the data suggest that asymptomatic HU favors the polarization of an anti-inflammatory M2-like macrophage phenotype that is associated with mitochondrial changes in morphology, e.g., fusion [55], for increased FAO but lower ROS, IL-6, and IL-1β production (Figure 1, Figure 2 and Figure S1). 

### 3.5. Asymptomatic Hyperuricemia Reduces Interstitial Fibrosis in Acute Organ Injury to Chronic Kidney Disease Transition

**Figure 7:**
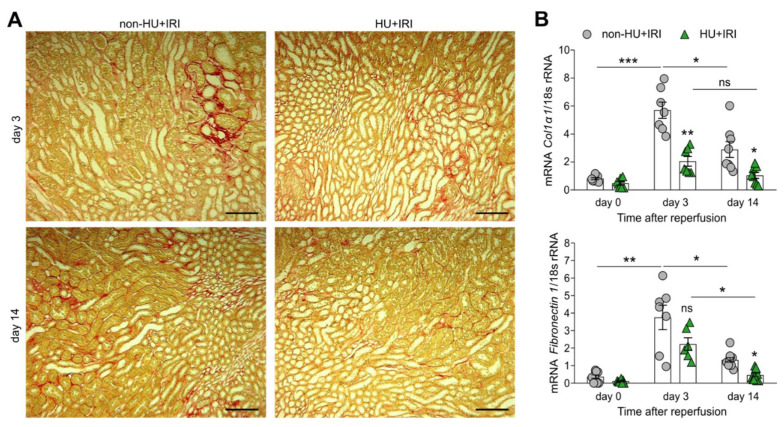
Asymptomatic hyperuricemia reduces interstitial fibrosis in acute organ injury. Hyperuricemic Alb-creERT2;*Glut9*^lox/lox^ (HU) and *Glut9*^lox/lox^ control (non-HU) mice were injected with tamoxifen and placed on a chow diet enriched with inosine to induce hyperuricemia (HU). Nephrectomy was performed (day 7) prior to clamping of the left renal pedicle for 15 min to induce ischemia/reperfusion injury (IRI). Mice were sacrificed on day 3 or 14. (**A**) Interstitial fibrosis illustrated on Picro-sirius red-stained kidney sections in non-HU and HU mice 3 and 14 days after IRI. Magnification, ×100. (**B**) Intrarenal mRNA expression levels of the fibrosis markers Col1α1 and fibronectin 1 in both groups of mice determined by RT-qPCR. Data are mean ± SEM from 7 mice per group. * *p* < 0.05; ** *p* < 0.01; *** *p* < 0.001; ns, not significant; two-way ANOVA with Tukey’s post hoc test.

Finally, to investigate whether asymptomatic HU influences interstitial fibrosis, we performed Picro sirius-red staining of kidney sections of non-HU and HU mice after IRI, and found that HU mice (HU + IRI) had fewer fibrotic lesions as compared with non-HU mice (non-HU + IRI) on day 3 and 14 (Figure 7A). Intrarenal mRNA expression levels of the fibrosis markers collagen (Col)1α1 and fibronectin 1 were significantly lower in HU mice after IRI on day 3 and 14, as compared with non-HU mice (Figure 7B). Thus, asymptomatic HU improves AKI–CKD transition by reducing tubular injury, inflammation, and interstitial fibrosis. 

## 4. Discussion

We had hypothesized that asymptomatic HU would elicit renoprotective effects during AKI/AKD because sUA can act as an anti-oxidant by enhancing metabolic activity. Furthermore, sUA might drive an anti-inflammatory M2-like macrophage phenotype to prevent further immunopathology and promote restoring of tubular epithelial cell integrity, thus improving the recovery after AKI/AKD. Indeed, our in vitro and in vivo data verify this hypothesis and highlight an anti-oxidative role for asymptomatic HU during acute organ injury.

Several in vitro studies reported that UA triggers the release of pro-inflammatory mediators, including NLRP3 inflammasome-mediated IL-1β production in lipopolysaccharide (LPS) and monosodium urate (MSU) crystal-stimulated murine and human monocytes and macrophages [17,18,19,20,21]. However, such studies prepared UA by pre-warming UA powder at 37 °C in culture medium, a preparation method that does not fully solubilize UA so that UA microcrystals remain as contaminants. Thus, “sUA” prepared by pre-warming UA solution triggers a pro-inflammatory response due to microcrystal contaminants identical to MSU crystal preparations [58,59], while a microcrystal-free UA solution prepared with sodium hydroxide rather reveals immunomodulatory effects on human CD14+ blood monocytes [25]. Here we now extend these findings and show that sUA also suppresses the pro-inflammatory functions, including ROS, IL-6, and IL-1β production of macrophages under hypoxic/inflammatory conditions, while enhancing FAO-related gene expression. Thus, sUA acts as antioxidant and contributes to the polarization of an anti-inflammatory M2-like macrophage phenotype via metabolic reprogramming. Soluble UA can scavenge ROS under hydrophilic conditions, thereby inhibiting lipid peroxidation [53]. Recent evidence suggests that xanthine oxidase activation but not sUA induces ROS and pro-inflammatory cytokine release in human macrophages [60]. Due to its anti-oxidative property, sUA is neuroprotective in Parkinson’s disease [61] and currently under clinical investigation to attenuate ischemic stroke [62,63].

So far, conflicting epidemiological and experimental data exist on the effects of HU in AKI/AKD. For example, epidemiological studies showed that elevated serum UA levels are associated with more severe AKI outcomes and/or a higher number of AKI episodes. However, they did not consider that an increased risk for AKI in HU patients could also be due to other AKI risk factors, such as obesity, high blood pressure, and chronic kidney disease [64]. Few clinical trials investigated the effect of so-called “urate lowering therapy” in AKI; however, for results obtained with xanthine oxidase inhibitors, it remains unclear whether these relate to inhibition of ROS or UA production [65,66]. Studies with specific modulation of UA, e.g., with recombinant uricase, are available only for acute urate nephropathy, which does represent the prevalent forms of AKI. Furthermore, pre-clinical studies investigating the impact of HU in AKI/AKD have very limited application to HU in humans because such animal models are either based on i.p. injections of high concentrations of UA [67,68,69], oral administration of potassium oxonate [70,71,72], or surgery-induced or diabetic nephropathy [18,20], inducing only very low serum UA levels (2–5 mg/dL) [16,73]. Using a novel animal model of asymptomatic HU with serum UA levels between 7 and 10 mg/dL that mimics the range of HU in humans more accurately [26], we now show that asymptomatic HU diminishes acute tubular injury, inflammation, and interstitial fibrosis, resulting in a better recovery 14 days after IRI. This is in line with recent data showing that asymptomatic HU does not induce kidney injury nor contribute to the progression of chronic kidney disease in mice [27]. Proof of concept comes from large multi-center randomized controlled trials on urate lowering therapy [74], while HU associated with UA crystalluria, i.e., symptomatic HU, causes acute and chronic kidney disease [13,27]. 

Alterations in cell energy metabolism, including glycolytic switch (defective FAO) and mitochondrial dysfunction, occur in animal models of AKI/AKD [75,76,77]. Although these changes could be components of physiologic metabolic variations in regenerating the epithelium, they could become pathologic if not reversed. For example, excessive mitochondrial fission (fragmented mitochondria), as seen in AKI/AKD, is associated with decreased FAO and reduced ATP production contributing to oxidative stress and injury in proximal tubules [78]. Consistent with our in vivo data, persistent upregulation of mitochondrial fission-related genes and downregulation of fusion-related genes have been overserved in tubular epithelial cells [79] even after kidney function is restored. Perry et al. reported that Drp1 mediates mitochondrial dysfunction and strengthens maladaptive repair processes, leading to fibrosis in an IR-induced murine model [80]. Mitochondrial fusion on the other hand mitigates the number of damaged mitochondria by combining their contents with healthy mitochondria through complementation, which contributes to increased FAO [78]. Knockout of Mfn2 in proximal tubules has been shown to accelerate recovery after AKI in mice [81]. Thus, highlighting the importance of maintaining mitochondrial dynamics and metabolism. Information on the role of FAO and mitochondrial dynamics in macrophages after AKI/AKD is limited. Our data now suggest that asymptomatic HU shifts the metabolic activity towards FAO, which drives M2-like macrophage polarization, thus contributing to the recovery of mice with AKI/AKD. This is consistent with reports showing that increased FAO [6,82] and mitochondrial dynamics [10] not only in macrophages but also in tubular epithelial cells can prevent tubular injury. Whether sUA and asymptomatic HU can enhance mitophagy in macrophages and tubular epithelial cells, a self-repair process to maintain mitochondrial homeostasis after acute organ injury [83], is currently unknown. Therapeutic interventions to specifically target sUA with urate depleting therapy, such as rasburicase or probenecid, are needed to answer this question and to confirm the potential anti-oxidative effect of asymptomatic HU during acute organ injury.

## 5. Conclusions

In conclusion, we found sUA to have anti-oxidative effects during acute organ injury. First, sUA reduced ROS and IL-6 production in macrophages, while increasing FAO under inflammatory and hypoxic conditions in vitro. Thus, sUA drives an anti-inflammatory M2-like macrophage phenotype via metabolic reprograming. Second, we verified in vivo that asymptomatic HU diminishes tubular injury, inflammation and interstitial fibrosis in a murine model of IRI. In particular, asymptomatic HU restored tubular integrity, enhanced mitochondrial dynamics and FAO, and contributed to anti-inflammatory M2-like macrophage polarization after IRI, thus improving the recovery after acute organ injury. Currently, no recommendation exists for the treatment of asymptomatic HU in AKI/AKD [84]. It is tempting to speculate that moderate serum UA levels should be tolerated during acute organ injury. Further investigations on the use of urate depleting therapy in this context are needed.

## Supplementary Materials

The following are available online at https://www.mdpi.com/article/10.3390/cells11040626/s1, Supplementary Table S1: Mouse primer sequences, Supplementary Figure S1: Soluble uric acid inhibits the pro-inflammatory function of LPS-activated macrophages, Supplementary Figure S2: Asymptomatic hyperuricemia does not induce endothelial dysfunction after acute organ injury, Supplementary Figure S3: Mitochondria gene expression profile of the kidney from mice after acute organ injury, Supplementary Figure S4: Asymptomatic hyperuricemia promotes mitochondria elongation in macrophages after acute organ injury.

## References

[1] Hoste E.A.J., Kellum J.A., Selby N.M., Zarbock A., Palevsky P.M., Bagshaw S.M., Goldstein S.L., Cerda J., Chawla L.S. (2018). Global epidemiology and outcomes of acute kidney injury. Nat. Rev. Nephrol..

[2] Kellum J.A., Romagnani P., Ashuntantang G., Ronco C., Zarbock A., Anders H.J. (2021). Acute kidney injury. Nat. Rev. Dis. Primers..

[3] Bonventre J.V., Yang L. (2011). Cellular pathophysiology of ischemic acute kidney injury. J. Clin. Investig..

[4] Suarez-Alvarez B., Liapis H., Anders H.J. (2016). Links between coagulation, inflammation, regeneration, and fibrosis in kidney pathology. Lab. Invest..

[5] Anders H.J. (2014). Immune system modulation of kidney regeneration-mechanisms and implications. Nat. Reviews. Nephrol..

[6] He L., Wei Q., Liu J., Yi M., Liu Y., Liu H., Sun L., Peng Y., Liu F., Venkatachalam M.A. (2017). AKI on CKD: Heightened injury, suppressed repair, and the underlying mechanisms. Kidney Int..

[7] Van den Bossche J., O’Neill L.A., Menon D. (2017). Macrophage Immunometabolism: Where Are We (Going)?. Trends Immunol..

[8] Huang S.C., Smith A.M., Everts B., Colonna M., Pearce E.L., Schilling J.D., Pearce E.J. (2016). Metabolic Reprogramming Mediated by the mTORC2-IRF4 Signaling Axis Is Essential for Macrophage Alternative Activation. Immunity.

[9] Pearce E.L., Pearce E.J. (2013). Metabolic pathways in immune cell activation and quiescence. Immunity.

[10] Szeto H.H. (2017). Pharmacologic Approaches to Improve Mitochondrial Function in AKI and CKD. J. Am. Soc. Nephrol..

[11] Shimada M., Johnson R.J., May W.S., Lingegowda V., Sood P., Nakagawa T., Van Q.C., Dass B., Ejaz A.A. (2009). A novel role for uric acid in acute kidney injury associated with tumour lysis syndrome. Nephrol. Dial. Transplant..

[12] Galardy P.J., Hochberg J., Perkins S.L., Harrison L., Goldman S., Cairo M.S. (2013). Rasburicase in the prevention of laboratory/clinical tumour lysis syndrome in children with advanced mature B-NHL: A Children’s Oncology Group Report. Br. J. Haematol..

[13] Preitner F., Laverriere-Loss A., Metref S., Da Costa A., Moret C., Rotman S., Bazin D., Daudon M., Sandt C., Dessombz A. (2013). Urate-induced acute renal failure and chronic inflammation in liver-specific Glut9 knockout mice. Am. J. Physiol. Renal. Physiol..

[14] Sanchez-Lozada L.G., Tapia E., Santamaria J., Avila-Casado C., Soto V., Nepomuceno T., Rodriguez-Iturbe B., Johnson R.J., Herrera-Acosta J. (2005). Mild hyperuricemia induces vasoconstriction and maintains glomerular hypertension in normal and remnant kidney rats. Kidney Int..

[15] Sautin Y.Y., Nakagawa T., Zharikov S., Johnson R.J. (2007). Adverse effects of the classic antioxidant uric acid in adipocytes: NADPH oxidase-mediated oxidative/nitrosative stress. Am. J. Physiol. Cell Physiol..

[16] Roncal C.A., Mu W., Croker B., Reungjui S., Ouyang X., Tabah-Fisch I., Johnson R.J., Ejaz A.A. (2007). Effect of elevated serum uric acid on cisplatin-induced acute renal failure. Am. J. Physiol. Renal. Physiol..

[17] Braga T.T., Davanso M.R., Mendes D., de Souza T.A., de Brito A.F., Cruz M.C., Hiyane M.I., de Lima D.S., Nunes V., de Fatima Giarola J. (2021). Sensing soluble uric acid by Naip1-Nlrp3 platform. Cell Death Dis..

[18] Kim S.M., Lee S.H., Kim Y.G., Kim S.Y., Seo J.W., Choi Y.W., Kim D.J., Jeong K.H., Lee T.W., Ihm C.G. (2015). Hyperuricemia-induced NLRP3 activation of macrophages contributes to the progression of diabetic nephropathy. Am. J. Physiol. Renal. Physiol..

[19] Martinez-Reyes C.P., Manjarrez-Reyna A.N., Mendez-Garcia L.A., Aguayo-Guerrero J.A., Aguirre-Sierra B., Villalobos-Molina R., Lopez-Vidal Y., Bobadilla K., Escobedo G. (2020). Uric Acid Has Direct Proinflammatory Effects on Human Macrophages by Increasing Proinflammatory Mediators and Bacterial Phagocytosis Probably via URAT1. Biomolecules.

[20] Braga T.T., Forni M.F., Correa-Costa M., Ramos R.N., Barbuto J.A., Branco P., Castoldi A., Hiyane M.I., Davanso M.R., Latz E. (2017). Soluble Uric Acid Activates the NLRP3 Inflammasome. Sci. Rep..

[21] Crisan T.O., Cleophas M.C.P., Novakovic B., Erler K., van de Veerdonk F.L., Stunnenberg H.G., Netea M.G., Dinarello C.A., Joosten L.A.B. (2017). Uric acid priming in human monocytes is driven by the AKT-PRAS40 autophagy pathway. Proc. Natl. Acad. Sci. USA.

[22] Mulay S.R., Steiger S., Shi C., Anders H.J. (2020). A guide to crystal-related and nano- or microparticle-related tissue responses. FEBS J..

[23] Honarpisheh M., Foresto-Neto O., Desai J., Steiger S., Gomez L.A., Popper B., Boor P., Anders H.J., Mulay S.R. (2017). Phagocytosis of environmental or metabolic crystalline particles induces cytotoxicity by triggering necroptosis across a broad range of particle size and shape. Sci. Rep..

[24] Mulay S.R., Anders H.J. (2016). Crystallopathies. N. Engl. J. Med..

[25] Ma Q., Honarpisheh M., Li C., Sellmayr M., Lindenmeyer M., Bohland C., Romagnani P., Anders H.J., Steiger S. (2020). Soluble Uric Acid Is an Intrinsic Negative Regulator of Monocyte Activation in Monosodium Urate Crystal-Induced Tissue Inflammation. J. Immunol..

[26] Preitner F., Pimentel A., Metref S., Berthonneche C., Sarre A., Moret C., Rotman S., Centeno G., Firsov D., Thorens B. (2015). No development of hypertension in the hyperuricemic liver-Glut9 knockout mouse. Kidney Int..

[27] Sellmayr M., Hernandez Petzsche M.R., Ma Q., Kruger N., Liapis H., Brink A., Lenz B., Angelotti M.L., Gnemmi V., Kuppe C. (2020). Only Hyperuricemia with Crystalluria, but not Asymptomatic Hyperuricemia, Drives Progression of Chronic Kidney Disease. J. Am. Soc. Nephrol..

[28] Marschner J.A., Schafer H., Holderied A., Anders H.J. (2016). Optimizing Mouse Surgery with Online Rectal Temperature Monitoring and Preoperative Heat Supply. Effects on Post-Ischemic Acute Kidney Injury. PLoS ONE.

[29] Nakazawa D., Kumar S.V., Marschner J., Desai J., Holderied A., Rath L., Kraft F., Lei Y., Fukasawa Y., Moeckel G.W. (2017). Histones and Neutrophil Extracellular Traps Enhance Tubular Necrosis and Remote Organ Injury in Ischemic AKI. J. Am. Soc. Nephrol..

[30] Schreiber A., Shulhevich Y., Geraci S., Hesser J., Stsepankou D., Neudecker S., Koenig S., Heinrich R., Hoecklin F., Pill J. (2012). Transcutaneous measurement of renal function in conscious mice. Am. J. Physiol. Renal. Physiol..

[31] Steiger S., Grill J.F., Ma Q., Bauerle T., Jordan J., Smolle M., Bohland C., Lech M., Anders H.J. (2018). Anti-Transforming Growth Factor beta IgG Elicits a Dual Effect on Calcium Oxalate Crystallization and Progressive Nephrocalcinosis-Related Chronic Kidney Disease. Front. Immunol..

[32] Lech M., Avila-Ferrufino A., Allam R., Segerer S., Khandoga A., Krombach F., Garlanda C., Mantovani A., Anders H.J. (2009). Resident dendritic cells prevent postischemic acute renal failure by help of single Ig IL-1 receptor-related protein. J. Immunol..

[33] Mulay S.R., Thomasova D., Ryu M., Anders H.J. (2012). MDM2 (murine double minute-2) links inflammation and tubular cell healing during acute kidney injury in mice. Kidney Int..

[34] Han S.J., Jang H.S., Noh M.R., Kim J., Kong M.J., Kim J.I., Park J.W., Park K.M. (2017). Mitochondrial NADP(+)-Dependent Isocitrate Dehydrogenase Deficiency Exacerbates Mitochondrial and Cell Damage after Kidney Ischemia-Reperfusion Injury. J. Am. Soc. Nephrol..

[35] Lech M., Anders H.J. (2014). Expression profiling by real-time quantitative polymerase chain reaction (RT-qPCR). Methods Mol. Biol..

[36] Li N., Steiger S., Fei L., Li C., Shi C., Salei N., Schraml B.U., Zheng Z., Anders H.J., Lichtnekert J. (2021). IRF8-Dependent Type I Conventional Dendritic Cells (cDC1s) Control Post-Ischemic Inflammation and Mildly Protect Against Post-Ischemic Acute Kidney Injury and Disease. Front. Immunol..

[37] Mills E.L., Kelly B., Logan A., Costa A.S.H., Varma M., Bryant C.E., Tourlomousis P., Dabritz J.H.M., Gottlieb E., Latorre I. (2016). Succinate Dehydrogenase Supports Metabolic Repurposing of Mitochondria to Drive Inflammatory Macrophages. Cell.

[38] Leal-Calvo T., Martins B.L., Bertoluci D.F., Rosa P.S., de Camargo R.M., Germano G.V., Brito de Souza V.N., Pereira Latini A.C., Moraes M.O. (2021). Large-Scale Gene Expression Signatures Reveal a Microbicidal Pattern of Activation in Mycobacterium leprae-Infected Monocyte-Derived Macrophages With Low Multiplicity of Infection. Front. Immunol..

[39] Askovich P.S., Ramsey S.A., Diercks A.H., Kennedy K.A., Knijnenburg T.A., Aderem A. (2017). Identifying novel transcription factors involved in the inflammatory response by using binding site motif scanning in genomic regions defined by histone acetylation. PLoS ONE.

[40] Lazzeri E., Angelotti M.L., Peired A., Conte C., Marschner J.A., Maggi L., Mazzinghi B., Lombardi D., Melica M.E., Nardi S. (2018). Endocycle-related tubular cell hypertrophy and progenitor proliferation recover renal function after acute kidney injury. Nat. Commun..

[41] Dobin A., Davis C.A., Schlesinger F., Drenkow J., Zaleski C., Jha S., Batut P., Chaisson M., Gingeras T.R. (2013). STAR: Ultrafast universal RNA-seq aligner. Bioinformatics.

[42] Lun A.T., McCarthy D.J., Marioni J.C. (2016). A step-by-step workflow for low-level analysis of single-cell RNA-seq data with Bioconductor. F1000Res.

[43] Haghverdi L., Lun A.T.L., Morgan M.D., Marioni J.C. (2018). Batch effects in single-cell RNA-sequencing data are corrected by matching mutual nearest neighbors. Nat. Biotechnol..

[44] Ke X., Chen C., Song Y., Cai Q., Li J., Tang Y., Han X., Qu W., Chen A., Wang H. (2019). Hypoxia modifies the polarization of macrophages and their inflammatory microenvironment, and inhibits malignant behavior in cancer cells. Oncol. Lett..

[45] Delprat V., Tellier C., Demazy C., Raes M., Feron O., Michiels C. (2020). Cycling hypoxia promotes a pro-inflammatory phenotype in macrophages via JNK/p65 signaling pathway. Sci. Rep..

[46] Diaz-Bulnes P., Saiz M.L., Lopez-Larrea C., Rodriguez R.M. (2019). Crosstalk Between Hypoxia and ER Stress Response: A Key Regulator of Macrophage Polarization. Front. Immunol..

[47] Tran M., Tam D., Bardia A., Bhasin M., Rowe G.C., Kher A., Zsengeller Z.K., Akhavan-Sharif M.R., Khankin E.V., Saintgeniez M. (2011). PGC-1alpha promotes recovery after acute kidney injury during systemic inflammation in mice. J. Clin. Investig..

[48] Svensson K., Schnyder S., Cardel B., Handschin C. (2016). Loss of Renal Tubular PGC-1alpha Exacerbates Diet-Induced Renal Steatosis and Age-Related Urinary Sodium Excretion in Mice. PLoS ONE.

[49] Mulay S.R., Honarpisheh M.M., Foresto-Neto O., Shi C., Desai J., Zhao Z.B., Marschner J.A., Popper B., Buhl E.M., Boor P. (2019). Mitochondria Permeability Transition versus Necroptosis in Oxalate-Induced AKI. J. Am. Soc. Nephrol..

[50] Tran M.T., Zsengeller Z.K., Berg A.H., Khankin E.V., Bhasin M.K., Kim W., Clish C.B., Stillman I.E., Karumanchi S.A., Rhee E.P. (2016). PGC1alpha drives NAD biosynthesis linking oxidative metabolism to renal protection. Nature.

[51] Jesinkey S.R., Funk J.A., Stallons L.J., Wills L.P., Megyesi J.K., Beeson C.C., Schnellmann R.G. (2014). Formoterol restores mitochondrial and renal function after ischemia-reperfusion injury. J. Am. Soc. Nephrol..

[52] Mulay S.R., Linkermann A., Anders H.J. (2016). Necroinflammation in Kidney Disease. J. Am. Soc. Nephrol..

[53] Ames B.N., Cathcart R., Schwiers E., Hochstein P. (1981). Uric acid provides an antioxidant defense in humans against oxidant- and radical-caused aging and cancer: A hypothesis. Proc. Natl. Acad. Sci. USA.

[54] Lech M., Grobmayr R., Weidenbusch M., Anders H.J. (2012). Tissues use resident dendritic cells and macrophages to maintain homeostasis and to regain homeostasis upon tissue injury: The immunoregulatory role of changing tissue environments. Mediat. Inflamm..

[55] Cervantes-Silva M.P., Cox S.L., Curtis A.M. (2021). Alterations in mitochondrial morphology as a key driver of immunity and host defence. EMBO Rep..

[56] Wang Y., Li N., Zhang X., Horng T. (2021). Mitochondrial metabolism regulates macrophage biology. J. Biol. Chem..

[57] Li Y., He Y., Miao K., Zheng Y., Deng C., Liu T.M. (2020). Imaging of macrophage mitochondria dynamics in vivo reveals cellular activation phenotype for diagnosis. Theranostics.

[58] Martinon F., Petrilli V., Mayor A., Tardivel A., Tschopp J. (2006). Gout-associated uric acid crystals activate the NALP3 inflammasome. Nature.

[59] Desai J., Steiger S., Anders H.J. (2017). Molecular Pathophysiology of Gout. Trends Mol. Med..

[60] Dai Y., Cao Y., Zhang Z., Vallurupalli S., Mehta J.L. (2017). Xanthine Oxidase Induces Foam Cell Formation through LOX-1 and NLRP3 Activation. Cardiovasc. Drugs Ther..

[61] Huang T.T., Hao D.L., Wu B.N., Mao L.L., Zhang J. (2017). Uric acid demonstrates neuroprotective effect on Parkinson’s disease mice through Nrf2-ARE signaling pathway. Biochem. Biophys. Res. Commun..

[62] Chamorro A., Amaro S., Castellanos M., Segura T., Arenillas J., Marti-Fabregas J., Gallego J., Krupinski J., Gomis M., Canovas D. (2014). Safety and efficacy of uric acid in patients with acute stroke (URICO-ICTUS): A randomised, double-blind phase 2b/3 trial. Lancet Neurol..

[63] Chamorro A., Amaro S., Castellanos M., Gomis M., Urra X., Blasco J., Arenillas J.F., Roman L.S., Munoz R., Macho J. (2017). Uric acid therapy improves the outcomes of stroke patients treated with intravenous tissue plasminogen activator and mechanical thrombectomy. Int. J. Stroke.

[64] Cheungpasitporn W., Thongprayoon C., Harrison A.M., Erickson S.B. (2016). Admission hyperuricemia increases the risk of acute kidney injury in hospitalized patients. Clin. Kidney J..

[65] Erol T., Tekin A., Katircibasi M.T., Sezgin N., Bilgi M., Tekin G., Zumrutdal A., Sezgin A.T., Muderrisoglu H. (2013). Efficacy of allopurinol pretreatment for prevention of contrast-induced nephropathy: A randomized controlled trial. Int. J. Cardiol..

[66] Kumar A., Bhawani G., Kumari N., Murthy K.S., Lalwani V., Raju Ch N. (2014). Comparative study of renal protective effects of allopurinol and N-acetyl-cysteine on contrast induced nephropathy in patients undergoing cardiac catheterization. J. Clin. Diagn. Res..

[67] Haryono A., Nugrahaningsih D.A.A., Sari D.C.R., Romi M.M., Arfian N. (2018). Reduction of Serum Uric Acid Associated with Attenuation of Renal Injury, Inflammation and Macrophages M1/M2 Ratio in Hyperuricemic Mice Model. Kobe J. Med. Sci..

[68] Romi M.M., Arfian N., Tranggono U., Setyaningsih W.A.W., Sari D.C.R. (2017). Uric acid causes kidney injury through inducing fibroblast expansion, Endothelin-1 expression, and inflammation. BMC Nephrol..

[69] Yang Z., Xiaohua W., Lei J., Ruoyun T., Mingxia X., Weichun H., Li F., Ping W., Junwei Y. (2010). Uric acid increases fibronectin synthesis through upregulation of lysyl oxidase expression in rat renal tubular epithelial cells. Am. J. Physiol. Renal. Physiol..

[70] Khosla U.M., Zharikov S., Finch J.L., Nakagawa T., Roncal C., Mu W., Krotova K., Block E.R., Prabhakar S., Johnson R.J. (2005). Hyperuricemia induces endothelial dysfunction. Kidney Int..

[71] Tung Y.T., Lin L.C., Liu Y.L., Ho S.T., Lin C.Y., Chuang H.L., Chiu C.C., Huang C.C., Wu J.H. (2015). Antioxidative phytochemicals from Rhododendron oldhamii Maxim. leaf extracts reduce serum uric acid levels in potassium oxonate-induced hyperuricemic mice. BMC Complement Altern. Med..

[72] Shi Y.W., Wang C.P., Wang X., Zhang Y.L., Liu L., Wang R.W., Ye J.F., Hu L.S., Kong L.D. (2012). Uricosuric and nephroprotective properties of Ramulus Mori ethanol extract in hyperuricemic mice. J. Ethnopharmacol..

[73] Patschan D., Patschan S., Gobe G.G., Chintala S., Goligorsky M.S. (2007). Uric acid heralds ischemic tissue injury to mobilize endothelial progenitor cells. J. Am. Soc. Nephrol..

[74] Steiger S., Ma Q., Anders H.J. (2020). The case for evidence-based medicine for the association between hyperuricaemia and CKD. Nat. Rev. Nephrol..

[75] Lan R., Geng H., Singha P.K., Saikumar P., Bottinger E.P., Weinberg J.M., Venkatachalam M.A. (2016). Mitochondrial Pathology and Glycolytic Shift during Proximal Tubule Atrophy after Ischemic AKI. J. Am. Soc. Nephrol..

[76] Kang H.M., Ahn S.H., Choi P., Ko Y.A., Han S.H., Chinga F., Park A.S., Tao J., Sharma K., Pullman J. (2015). Defective fatty acid oxidation in renal tubular epithelial cells has a key role in kidney fibrosis development. Nat. Med..

[77] Ash S.R., Cuppage F.E. (1970). Shift toward anaerobic glycolysis in the regenerating rat kidney. Am. J. Pathol..

[78] Bhargava P., Schnellmann R.G. (2017). Mitochondrial energetics in the kidney. Nat. Rev. Nephrol..

[79] Funk J.A., Schnellmann R.G. (2012). Persistent disruption of mitochondrial homeostasis after acute kidney injury. Am. J. Physiol. Renal. Physiol..

[80] Perry H.M., Huang L., Wilson R.J., Bajwa A., Sesaki H., Yan Z., Rosin D.L., Kashatus D.F., Okusa M.D. (2018). Dynamin-Related Protein 1 Deficiency Promotes Recovery from AKI. J. Am. Soc. Nephrol..

[81] Gall J.M., Wang Z., Bonegio R.G., Havasi A., Liesa M., Vemula P., Borkan S.C. (2015). Conditional knockout of proximal tubule mitofusin 2 accelerates recovery and improves survival after renal ischemia. J. Am. Soc. Nephrol..

[82] Fedorova L.V., Sodhi K., Gatto-Weis C., Puri N., Hinds T.D., Shapiro J.I., Malhotra D. (2013). Peroxisome proliferator-activated receptor delta agonist, HPP593, prevents renal necrosis under chronic ischemia. PLoS ONE.

[83] Tang C., Han H., Yan M., Zhu S., Liu J., Liu Z., He L., Tan J., Liu Y., Liu H. (2018). PINK1-PRKN/PARK2 pathway of mitophagy is activated to protect against renal ischemia-reperfusion injury. Autophagy.

[84] Chales G. (2019). How should we manage asymptomatic hyperuricemia?. Joint Bone Spine.

[85] Kilkenny C., Browne W.J., Cuthill I.C., Emerson M., Altman D.G. (2010). Improving bioscience research reporting: The ARRIVE guidelines for reporting animal research. PLoS Biol..

