# Supplementary material for: Asymptomatic Hyperuricemia Promotes Recovery from Ischemic Organ Injury by Modulating the Phenotype of Macrophages
Source: Cells. 2022 Feb 11;11(4):626. doi: 10.3390/cells11040626 (PMC8869798; doi:10.3390/cells11040626)
Supplement: Supplementary file 1 [file cells-11-00626-s001.zip › cells-1504332-supplementary.pdf]

cells MDPI

Supplementary Materials

Asymptomatic Hyperuricemia Promotes Recovery from Ischemic Organ Injury by Modulating the Phenotype of Macrophages

Viviane Gnemmi [1,2], Qiubo Li [2], Qiuyue Ma [2], Letizia De Chiara [3], Giulia Carangelo [3], Chenyu Li [2], Mireia Molina-Van den Bosch [2], Paola Romagnani [3], Hans-Joachim Anders [2] and Stefanie Steiger [2,*]

1 Service d'Anatomie Pathologique, Centre de Biologie Pathologique, CHU Lille, 59037 Lille, France; viviane.gnemmi@univ-lille.fr
2 Division of Nephrology, Department of Medicine IV, University Hospital, Ludwig-Maximilians-University Munich, 80336 Munich, Germany; qiubo.li@med.uni-muenchen.de (Q.L.); maqiuyue2014@hotmail.com (Q.M.); chenyu.li@med.uni-muenchen.de (C.L.); mireiamoli@gmail.com (M.M.-V.d.B.);hans-joachim.anders@med.uni-muenchen.de (H.-J.A.)
3 Department of Experimental and Clinical Biomedical Sciences "Mario Serio", University of Florence, 50139 Florence, Italy; letizia.dechiara@unifi.it (L.D.C.); giulia.carangelo@unifi.it (G.C.); paola.romagnani@unifi.it (P.R.)
* Correspondence: stefanie.steiger@med.uni-muenchen.de

Table of content

1. Supplementary Tables 2
    1.1 Supplementary Table S1 2
2. Supplementary Figures 3
    2.1 Supplementary Figure S1 3
    2.2 Supplementary Figure S2 4
    2.3 Supplementary Figure S3 5
    2.4 Supplementary Figure S4 6

Supplementary Table S1. Mouse primer sequences.

Gene name	Primer Sequences
KIM-1	Forward: TCAGCTCGGGAATGCACAA<br>Reverse: TGGTTGCCTTCCGTGTCTCT
Col1α1	Forward: ACATGTTCAGCTTTGTGGACC<br>Reverse: TAGGCCATTGTGTATGCAGC
Fibronectin 1	Forward: GGAGTGGCACTGTCAACCTC<br>Reverse: ACTGGATGGGGTGGGAAT
iNos	Forward: GAGACAGGGAAGTCTGAAGCAC<br>Reverse: CCAGCAGTAGTTGCTCCTCTTC
Il6	Forward: TGATGCACTTGCAGAAAACA<br>Reverse: ACCAGAGGAAATTTTCAATAGGC
Tnfα	Forward: AGGGTCTGGGCCATAGAACT<br>Reverse: CCACCACGCTCTTCTGTCTAC
Arginase 1/Arg1	Forward: AGAGATTATCGGAGCGCCTT<br>Reverse: TTTTTCCAGCAGACCAGCTT
Fizz-1	Forward: CCCTTCTCATCTGCATCTCC<br>Reverse: CTGGATTGGCAAGAAGTTCC
Ho-1	Forward: GAAATCATCCCTTGCACGCC<br>Reverse : CCTGAGAGGTCACCCAGGTA
Nrf-1	Forward: GCACCTTTGGAGAATGTGGT<br>Reverse: GATAAATGCCCGAAGCTGAG
Sod	Forward: CCAGTGCAGGACCTCATTTT<br>Reverse: GTTTACTGCGCAATCCCAAT
Cpt1	Forward: CTCCGCCTGAGCCATGAAG<br>Reverse: CACCAGTGATGATGCCATTCT
Pparg	Forward: GGAAGACCACTCGCATTCCTT<br>Reverse: GTAATCAGCAACCATTGGGTCA
Pgc1b	Forward: TCCTGTAAAAGCCCGGAGTAT<br>Reverse: GCTCTGGTAGGGGCAGTGA
Glut1	Forward: GCCTGACCTTCGGATATGAGC<br>Reverse: TGCCATAGCAGTCAATGAGGA
Slc2a9/Glut9	Forward: CTGTCCAGATGTTGTCTAGG<br>Reverse: GTTATGATGCAGGAGCTTAGC
Drp1	Forward: GGGCACTTAAATTGGGCTCC<br>Reverse: TGTATTCTGTTGGCGTGGAAC
Fis1	Forward: GGCTGTCTCCAAGTCCAAATC<br>Reverse: GGAGAAAAGGGAAGGCGATG
Dnm1	Forward: GTGGACATGGTTATCTCGGAGC<br>Reverse: GGTGGTCACAATTCGCTCCATC
Opa1	Forward: TCACCTCTGCGTTTATTTGAAGA<br>Reverse: GGGTAGAACGGAGGAAAGG
Mfn1	Forward: TATCGATGCCTTGCGGAGAT<br>Reverse: GGCGAATCACAACACTTCCA
Mfn2	Forward: GGAGACCAACAAGGACTGGA<br>Reverse: TGCACAGTGACTTTCAACCG
18s	Forward: GCAATTATTCCCCATGAACG<br>Reverse: AGGGCCTCACTAAACCATCC

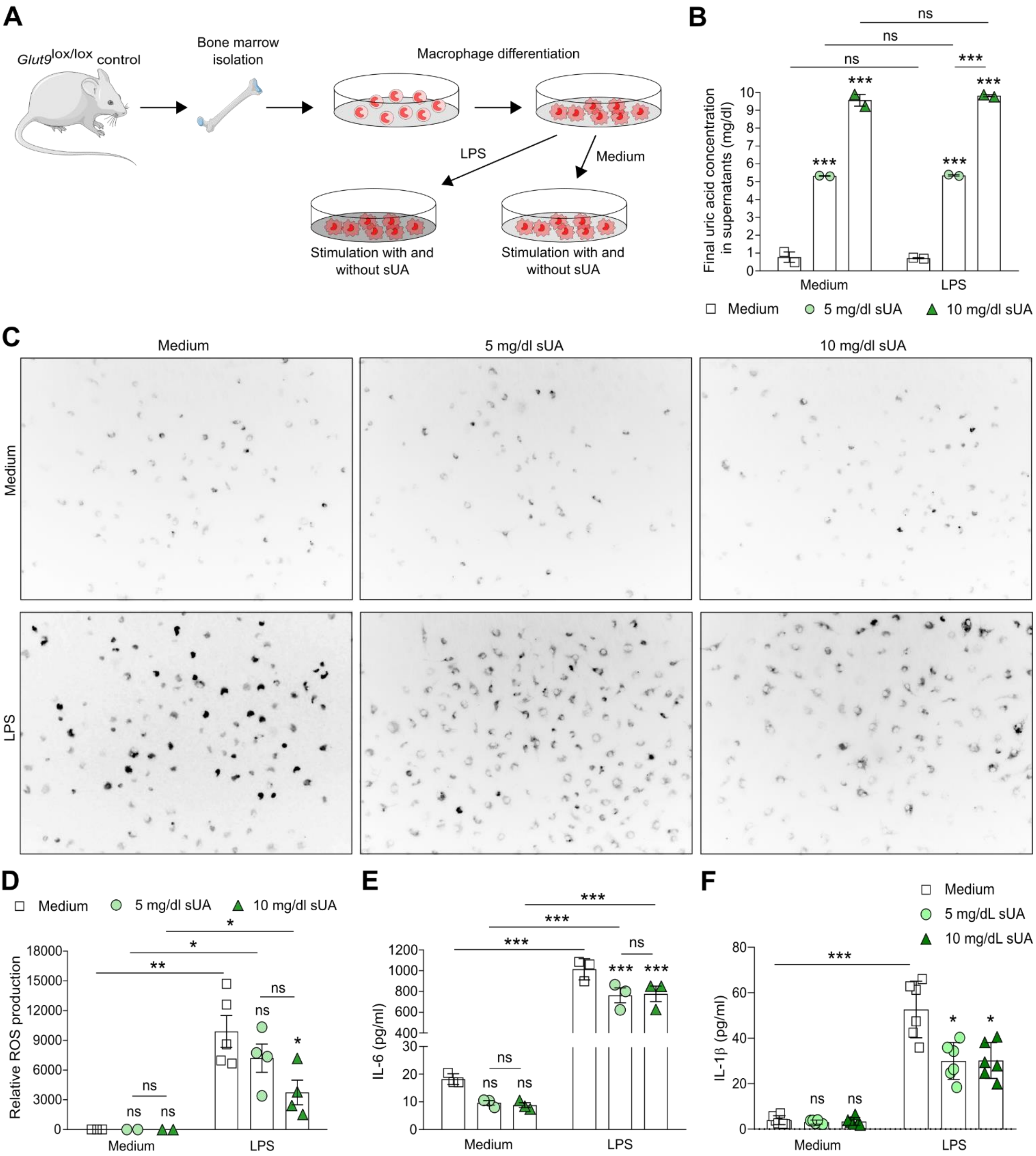

Supplementary Figure S1. Soluble uric acid inhibits the pro-inflammatory function of LPS-activated macrophages. (**A**) Schematic of experimental setup. Bone marrow–derived macrophages from *Glut9*$^{lox/lox}$ mice were primed with LPS for 24 h prior to sUA stimulation (medium; 5 mg/dL sUA; 10 mg/dL sUA). Afterwards, cells were stimulated with and without LPS (100 ng/mL) for 24 h. (**B**) Final UA concentrations after adding 5 and 10 mg/dL sUA into the macrophage culture were measured by colorimetric assay (n = 2). (**C**,**D**) Representative images (C) and quantification of ROS production (D) was detected with dihydrorhodamine 123 dye using a fluorescent microscope (n = 2-5). (**E**) Concentrations of interleukin (IL)-6 in culture supernatants were determined by ELISA (n = 3). (**F**) Concentration of interleukin (IL)-1β in culture supernatants were determined by ELISA (n = 6). Data are expressed as mean ± SEM. * $p<0.05$, ** $p<0.01$, *** $p<0.001$, ns, not significant; two-way ANOVA with Tukey post-test.

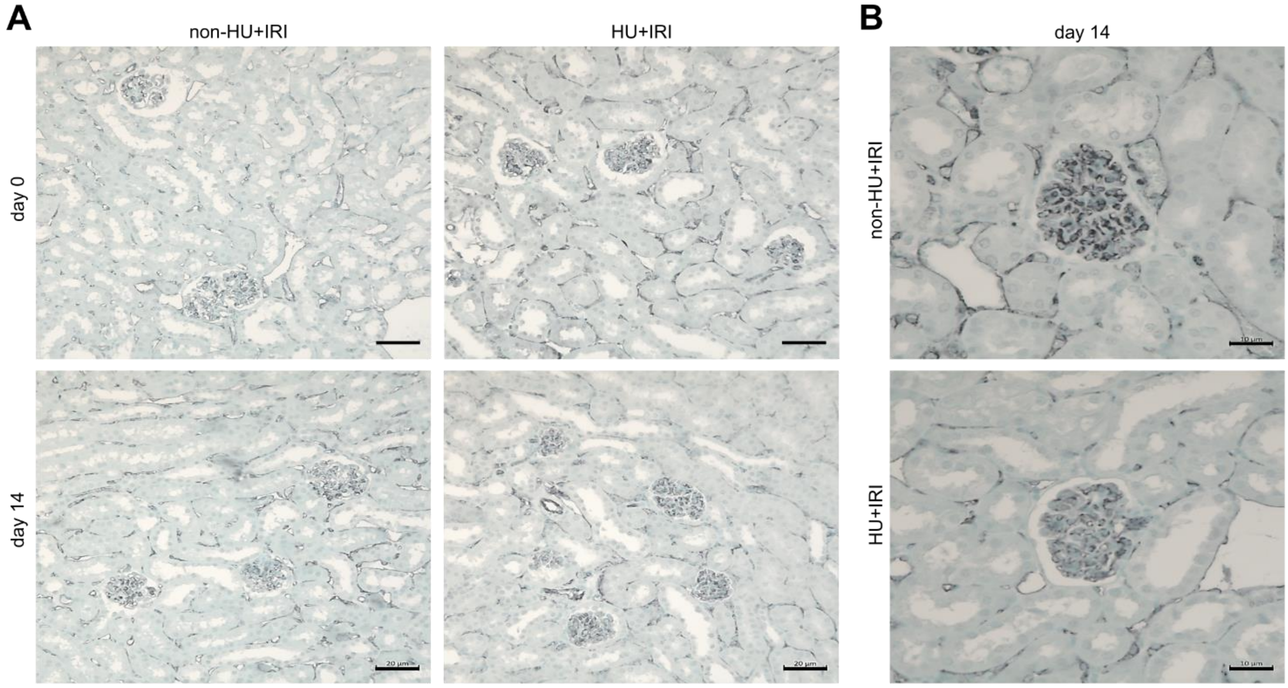

Supplementary Figure S2. Asymptomatic hyperuricemia does not induce endothelial dysfunction after acute organ injury. Hyperuricemic Alb-creERT2;*Glut9*$^{lox/lox}$ (HU) and *Glut9*$^{lox/lox}$ control (non-HU) mice were injected with tamoxifen and placed on a chow diet enriched with inosine to induce hyperuricemia (HU). Nephrectomy was performed (-day 7) prior to clamping of the left renal pedicle for 15 min to induce ischemia/reperfusion injury (IRI). Mice were sacrificed on day 14. (**A**,**B**) Representative images of CD31 staining of glomerular and peritubular endothelial cells on kidney sections in non-HU+IRI and HU+IRI mice on day 0 and 14. Magnification x200 (**A**) and x400 (**B**).

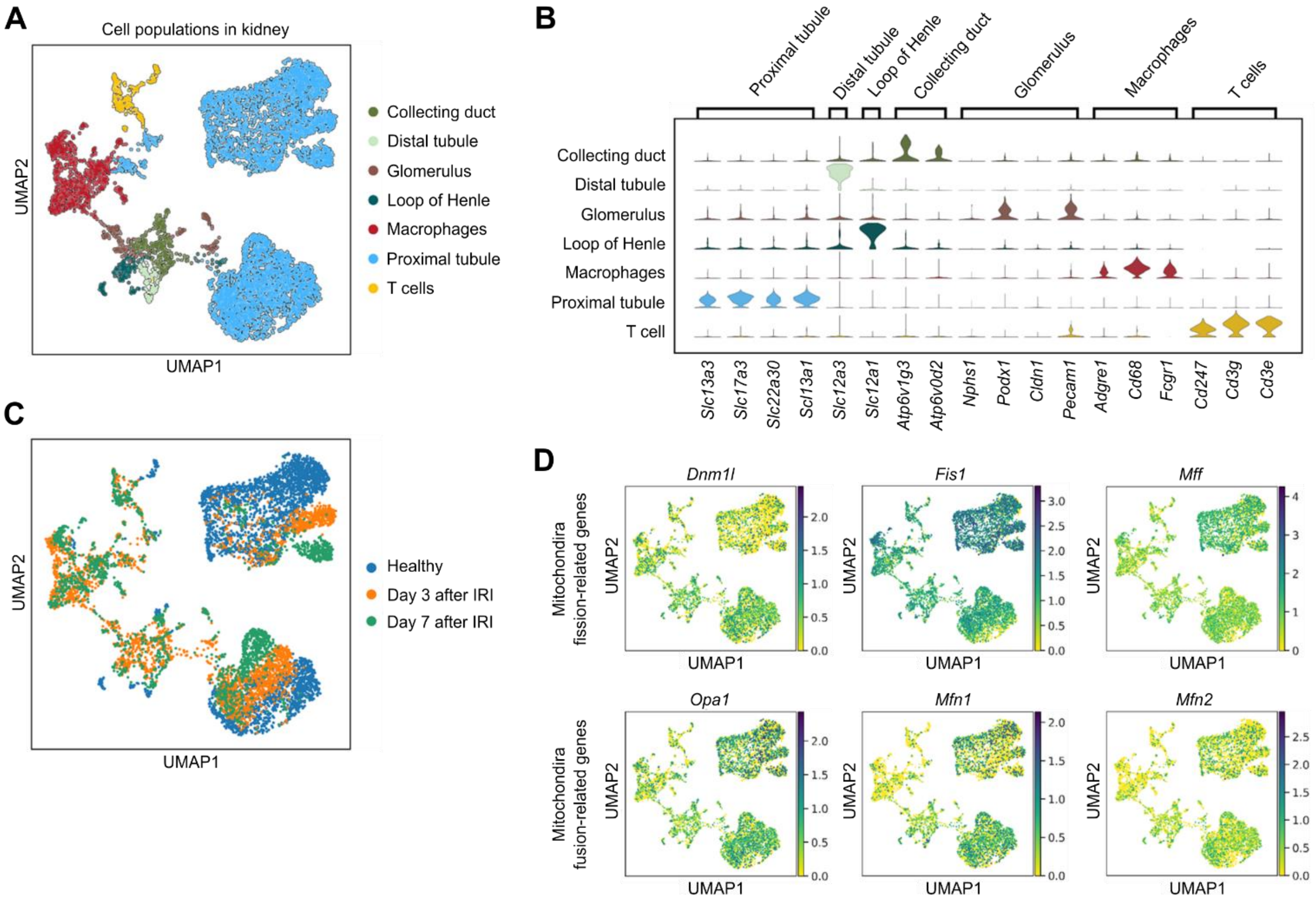

Supplementary Figure S3. Mitochondria gene expression profile of the kidney from mice after acute organ injury. Ischemia/reperfusion injury (IRI) was induced in wild-type C57Bl/6 mice by clamping the left renal pedicle for 30 min. Mice were sacrifice at 3 days (n = 3) and 7 days (n = 3) after IRI-induced AKI. Healthy mouse kidney (n = 1) was used as control. Single cell RNA sequencing of kidney samples was performed. (**A**) UMAP projection showing cluster distribution of mouse kidney cells. (**B**) Violin cluster characterization of cell population. (**C**) UMAP projection showing cluster distribution of mouse kidney cells in healthy mice and mice on day 3 and 7 after IRI-induced AKI. (**D**) UMAP projection showing cluster distribution of mitochondria fission- and fusion-related genes of mouse kidney cells.

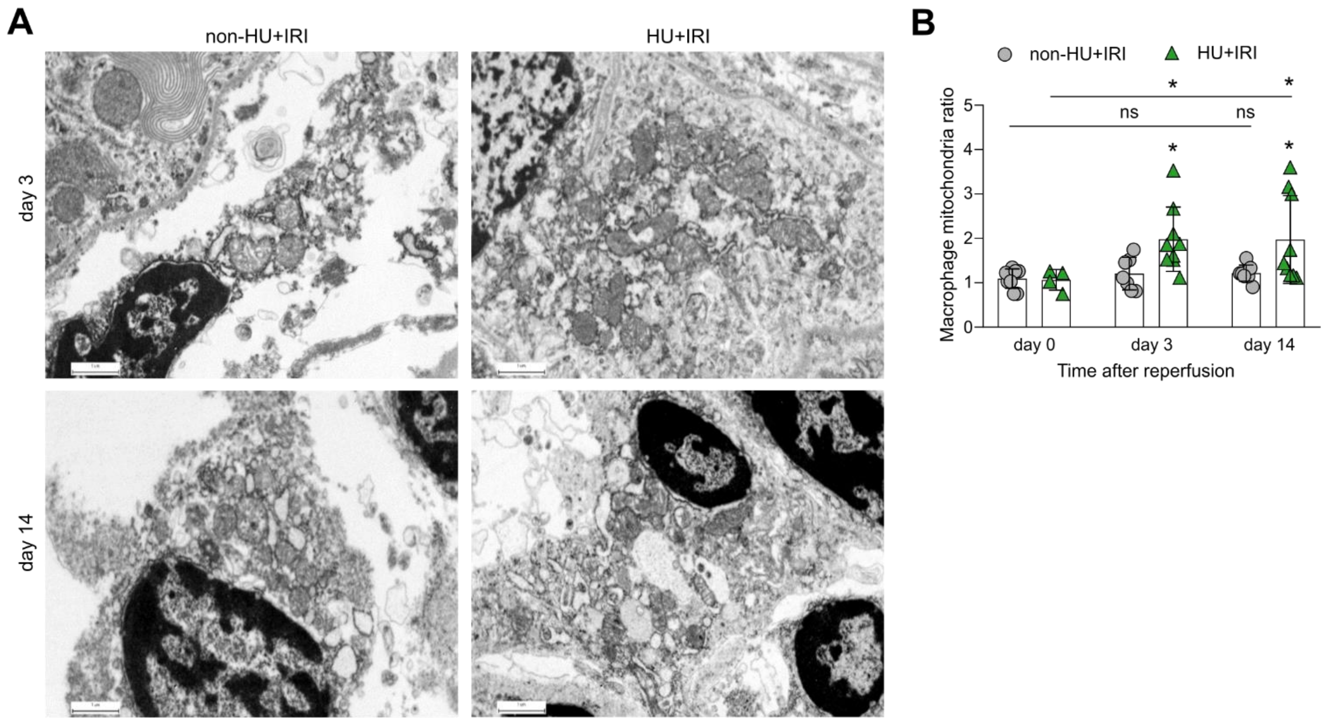

Supplementary Figure S4. Asymptomatic hyperuricemia promotes mitochondria elongation in macrophages after acute organ injury. Hyperuricemic Alb-creERT2;*Glut9*$^{lox/lox}$ (HU) and *Glut9*$^{lox/lox}$ control (non-HU) mice were injected with tamoxifen and placed on a chow diet enriched with inosine to induce hyperuricemia (HU). Nephrectomy was performed (day 7) prior to clamping of the left renal pedicle for 15 min to induce ischemia/reperfusion injury (IRI). Mice were sacrificed on day 3 or 14. (**A**,**B**) Electron microscopy of mitochondria morphology in macrophages (**A**) and quantification of the mitochondrial long axis/short axis ratio (n = 9-11 mitochondria, (**B**). Data are mean ± SEM from 2-3 mice per group. * $p<0.05$, ns, not significant; two-way ANOVA with Tukey's post-test.
